# Supplementary figures and images for: ST8Sia2 polysialyltransferase protects against infection by Trypanosoma cruzi
Source: PLoS Negl Trop Dis. 2024 Sep 25;18(9):e0012454. doi: 10.1371/journal.pntd.0012454 (PMC11466412; doi:10.1371/journal.pntd.0012454)

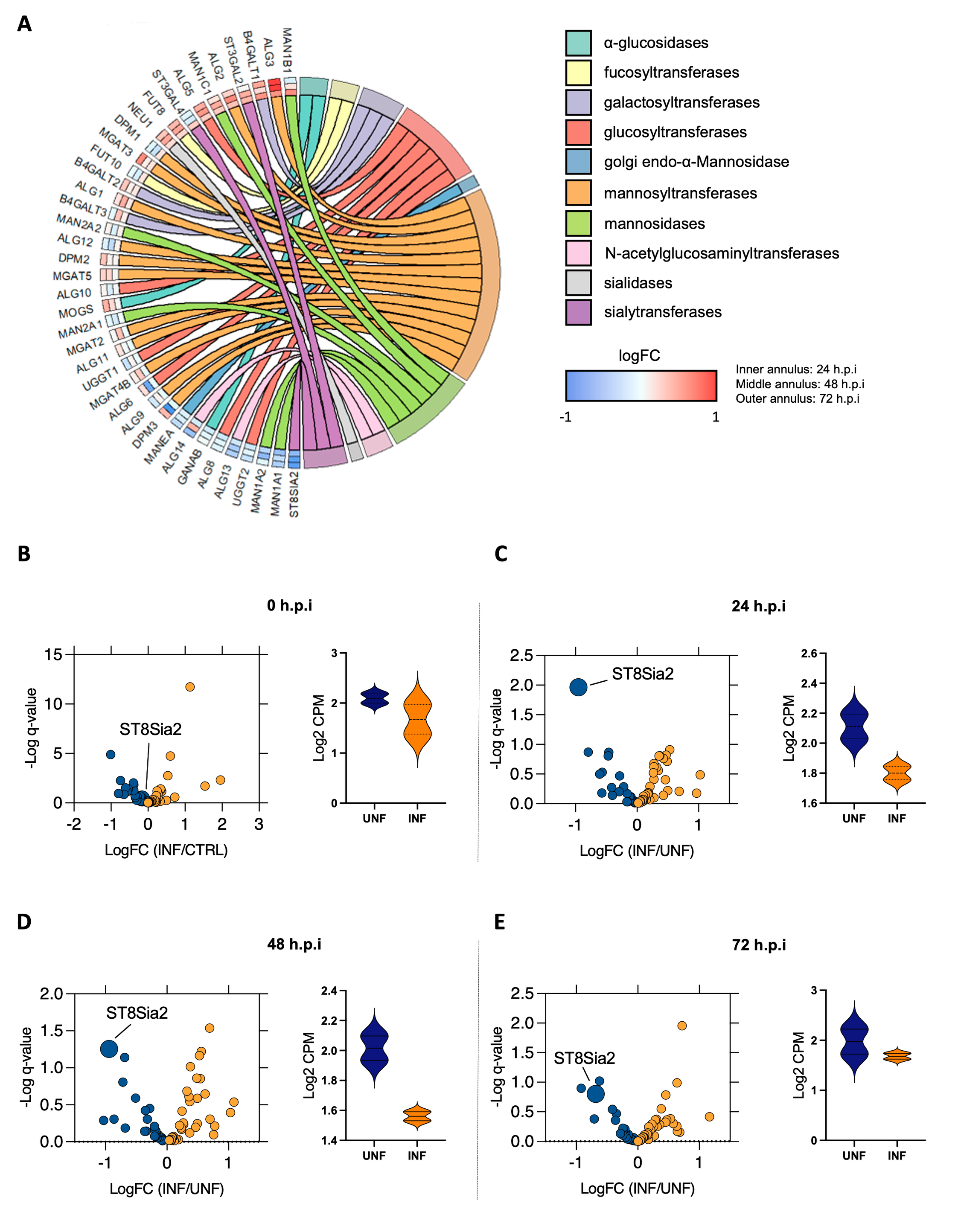

Supplement: S1 Fig — Bioinformatics analysis of Human induced pluripotent stem cell-derived cardiomyocytes (hiPSC-CM) infected with Trypanosoma cruzi (Y strain). (A) Chordplot indicating the modulation of N-glycosylation machinery transcripts at 24 h (inner), 48 h (middle), and 72 h (outer) post-infection (h.p.i) with T. cruzi (infected vs non-infected; INFvsUNF). The R GOplot package was used to build chord plot. Red and blue colors indicate upregulated and downregulated transcripts, respectively; (B-E) Volcano plot (left graph) and Violin plot (right graph) of transcripts encoding enzymes involved in the N-glycosylation pathway that were differentially regulated in T. cruzi-infected hiPSC-CM, focusing on the modulation of the ST8Sia2 transcripts after 0 h (B), 24 h (C), 48 h (D), and 72h (E) post-infection. The logFC (INFvsUNF) indicates transcripts upregulated in orange and downregulated in dark blue. (TIF) [file pntd.0012454.s001.tif]

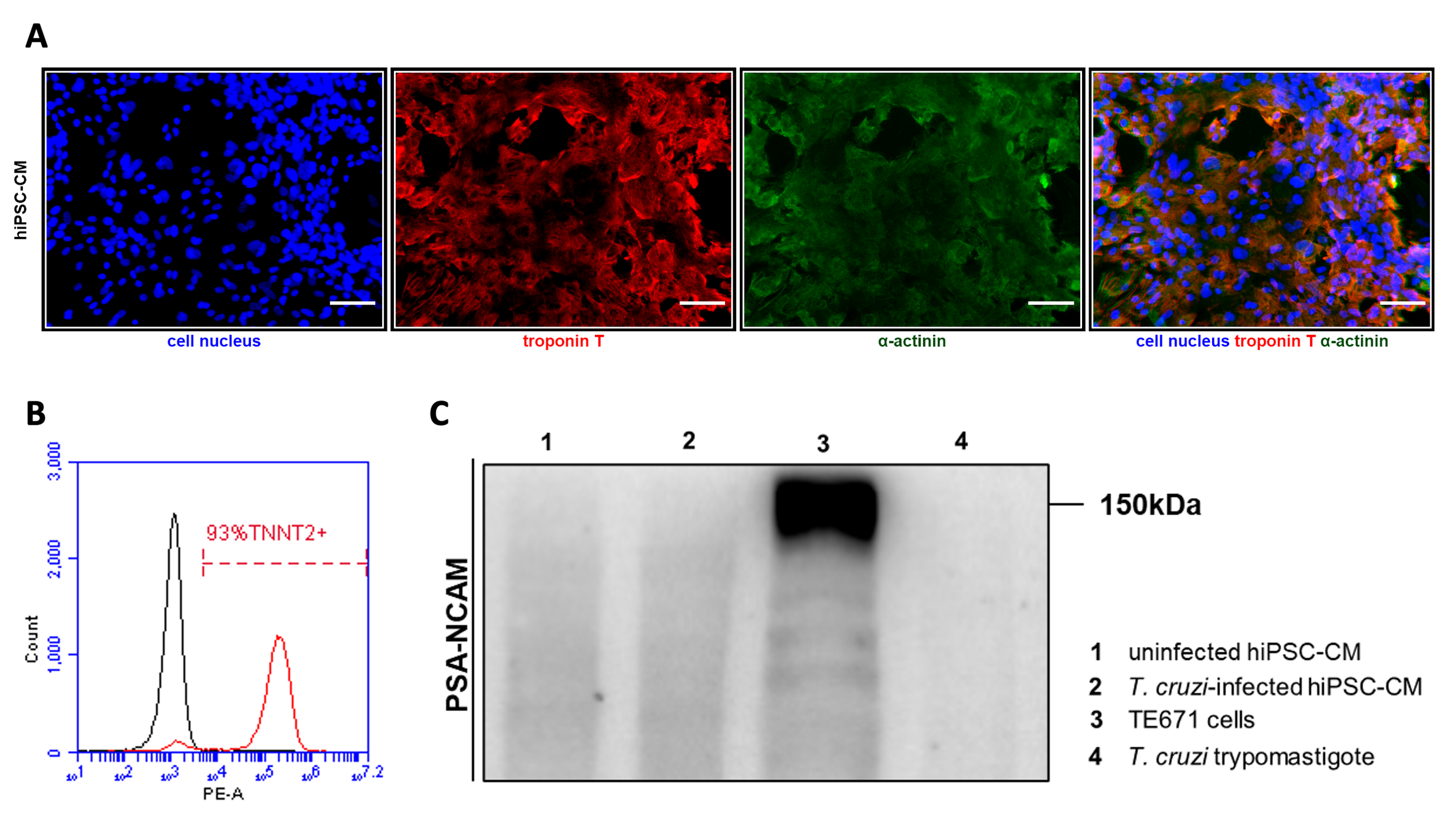

Supplement: S2 Fig — (A) Representative immunofluorescence images of hiPSC-CM confirming the presence of α-actinin and troponin T (sarcomeric proteins). Cell nucleus were stained with DAPI (scale bars = 10 μm). (B) Histogram of Troponin T (TNNT2+) staining the differentiation of hiPSC into hiPSC-CM. (C) Representative western blot images of PSA-NCAM levels in uninfected hiPSC-CM (lane 1), T. cruzi-infected hiPSC-CM (lane 2), TE671 cells (lane 3), and T. cruzi trypomastigote (lane 4). hiPSC-CM cells were seeded in 6-well microplates (1 x 10^6^ cells/well) and infected with T. cruzi trypomastigotes (Y strain) in a ratio of 1:5 (SH-SY5Y:trypomastigotes) for 48 h at 37° C. Protein extracts from TE671 cell lysates (in Laemmli buffer) and T. cruzi trypomastigote (in RIPA buffer) were used as a positive and negative control for PSA-NCAM, respectively. 15 μg of protein extracts were used to analyze the PSA-NCAM levels by Western blotting using a specific anti-PSA-NCAM antibody. (TIF) [file pntd.0012454.s002.tif]

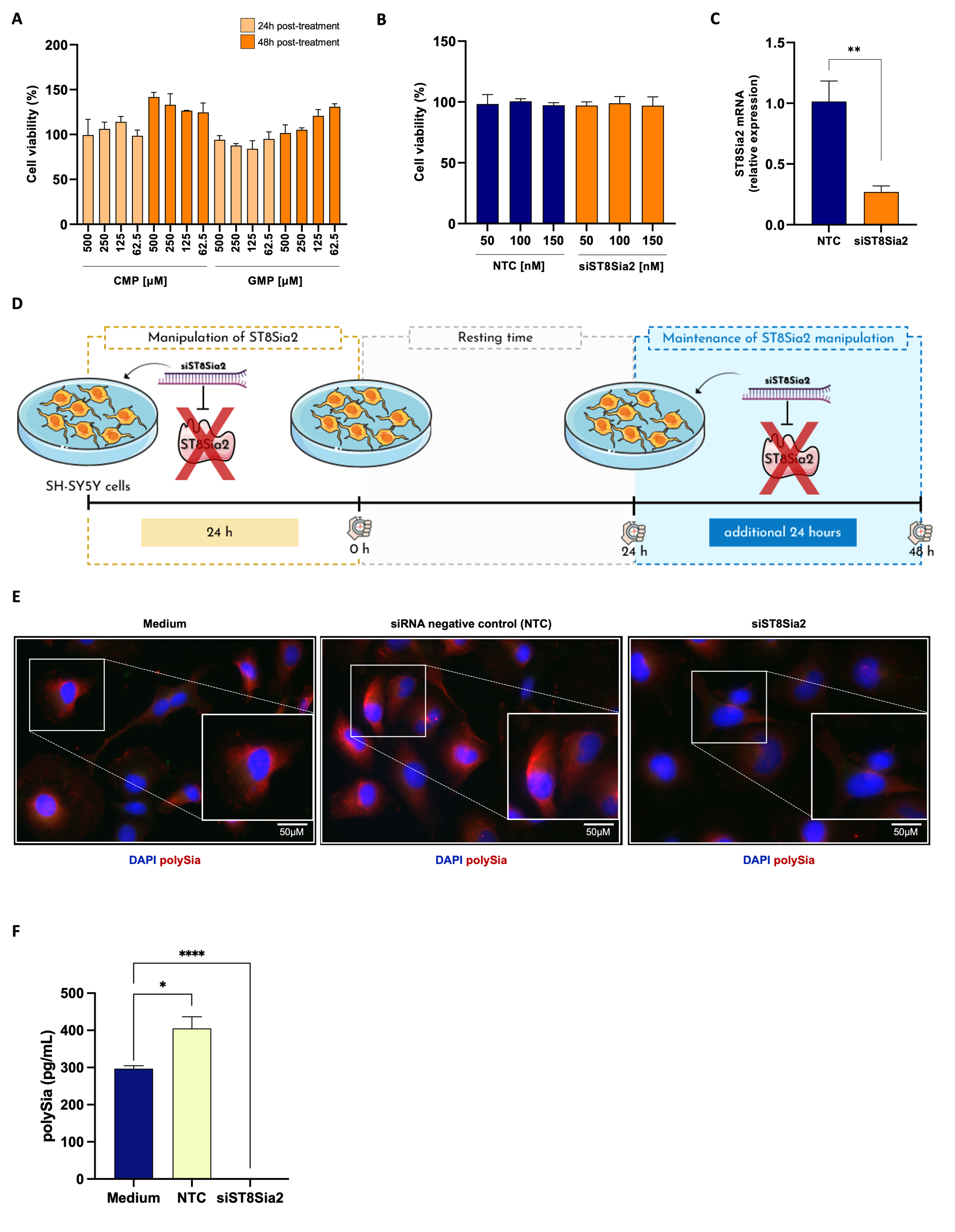

Supplement: S3 Fig — (A,B) SH-SY5Y cells were seeded in 96-well microplates (1 × 10^5^ cells/well), and treated with cytidine 5’-monophosphate (CMP; 62.5–500 μM) or siRNA ST8Sia2 (50–150 nM) for 24 and/or 48 h. Guanosine 5’-monophosphate (GMP), siRNA negative control (NTC), and/or medium alone were used as negative controls for chemical or genetic inhibition of ST8Sia2. In all controls used in experiments with siRNA was added Lipofectamine RNAiMAX. Following 24 and 48 h incubation with CMP (A) or 24 h incubation with siRNA ST8Sia2 (B), 3-(4,5-dimethyl-2-thiazolyl)-2,5-diphenyl-2H-tetrazolium bromide (MTT; 50 μg/mL) was added to the cells, and mitochondrial activity was estimated by MTT reduction and expressed as a percentage calculated from the ratio between the absorbance of stimulated and non-stimulated SH-SY5Y cells; (C) Relative expression of ST8Sia2 mRNA measured by qRT-PCR in SH-SY5Y cells treated with siRNA ST8Sia2 (siST8Sia2), following the experimental workflow presented in D. siRNA negative control (NTC) were used as negative control for genetic silencing of ST8Sia2. The Ct values of the target transcripts were normalized to the relative expression of GAPDH as endogenous control, and the relative expression of ST8Sia2 transcripts was quantified by the 2-ΔΔ Ct method. Each bar represents the mean of three independent experiments performed in triplicate; (D) Experimental workflow adopted to investigate the effect of genetic inhibition of ST8Sia2 in SH-SY5Y cells using 100 nM of siRNA ST8Sia2. SH-SY5Y cells were seeded in 24-well microplates (5 x 10^4^ cells/well), and treated with siRNA ST8Sia2 [100 nM] for 24h, followed by resting time of 24 h additional. After this resting time, siST8Sia2 SH-SY5Y cells were restimulated with siRNA ST8Sia2 [100 nM] for an additional 24h. siRNA negative control (NTC) were used as negative control. In all controls used in experiments with siRNA was added Lipofectamine RNAiMAX; (E) Representative images of polySia levels in siST8Sia2 SH-SY5Y cells. SH [file pntd.0012454.s003.tif]
