# Supplementary material for: ST8Sia2 polysialyltransferase protects against infection by Trypanosoma cruzi
Source: PLoS Negl Trop Dis. 2024 Sep 25;18(9):e0012454. doi: 10.1371/journal.pntd.0012454 (PMC11466412; doi:10.1371/journal.pntd.0012454)

S2A Fig

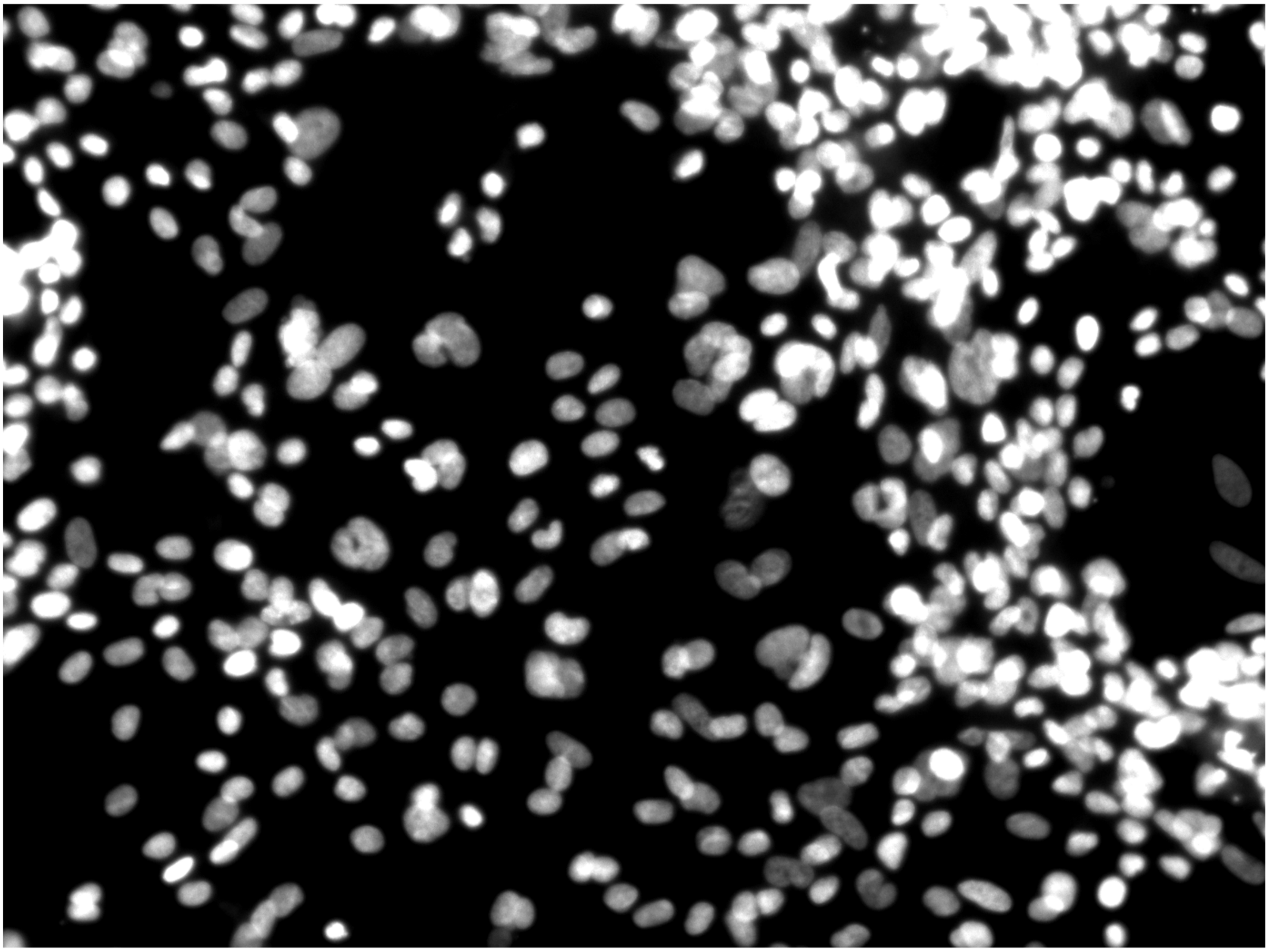

DAPI

S2A Fig

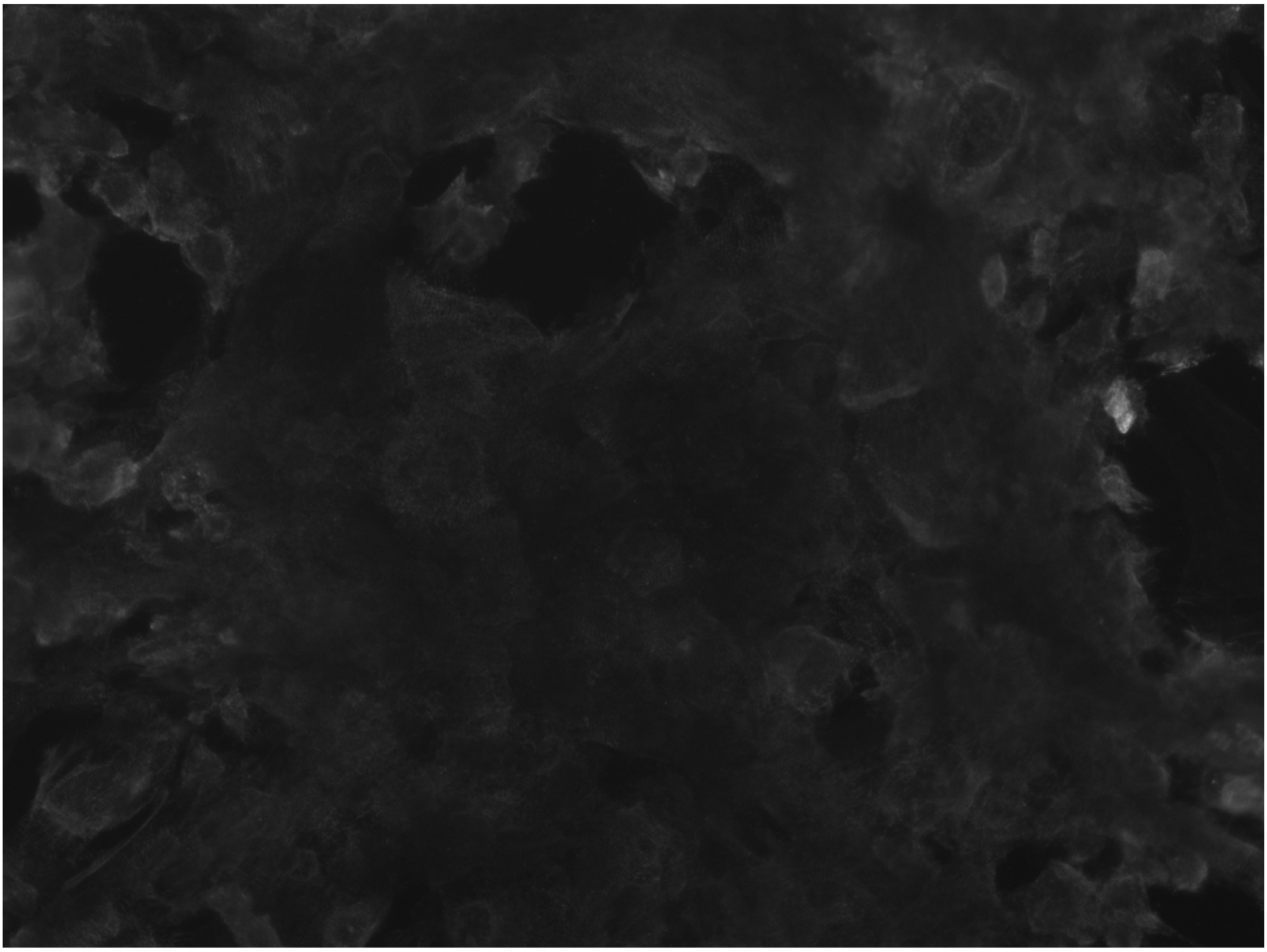

α-actinin

S2A Fig

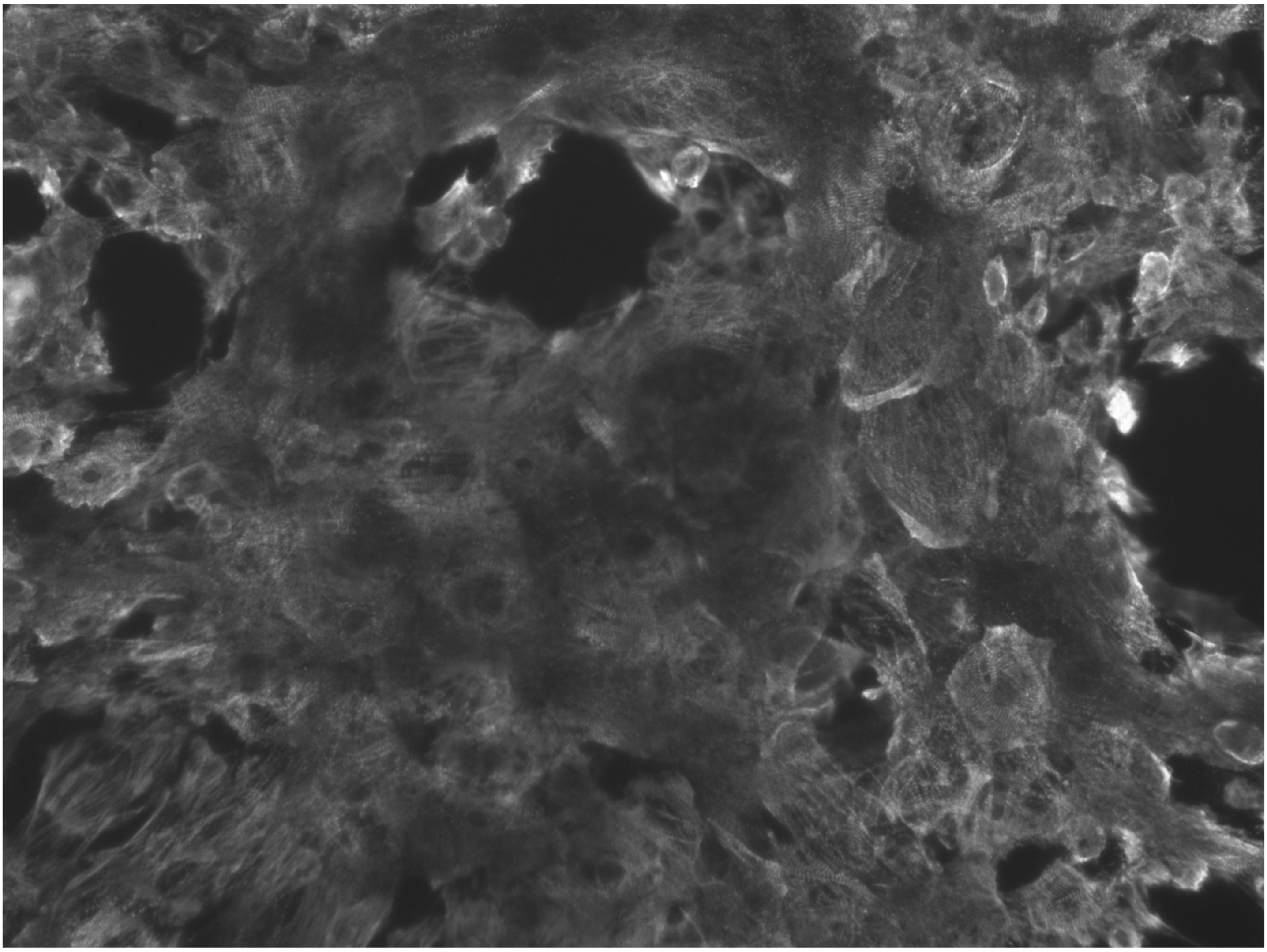

troponin T

Fig 1A

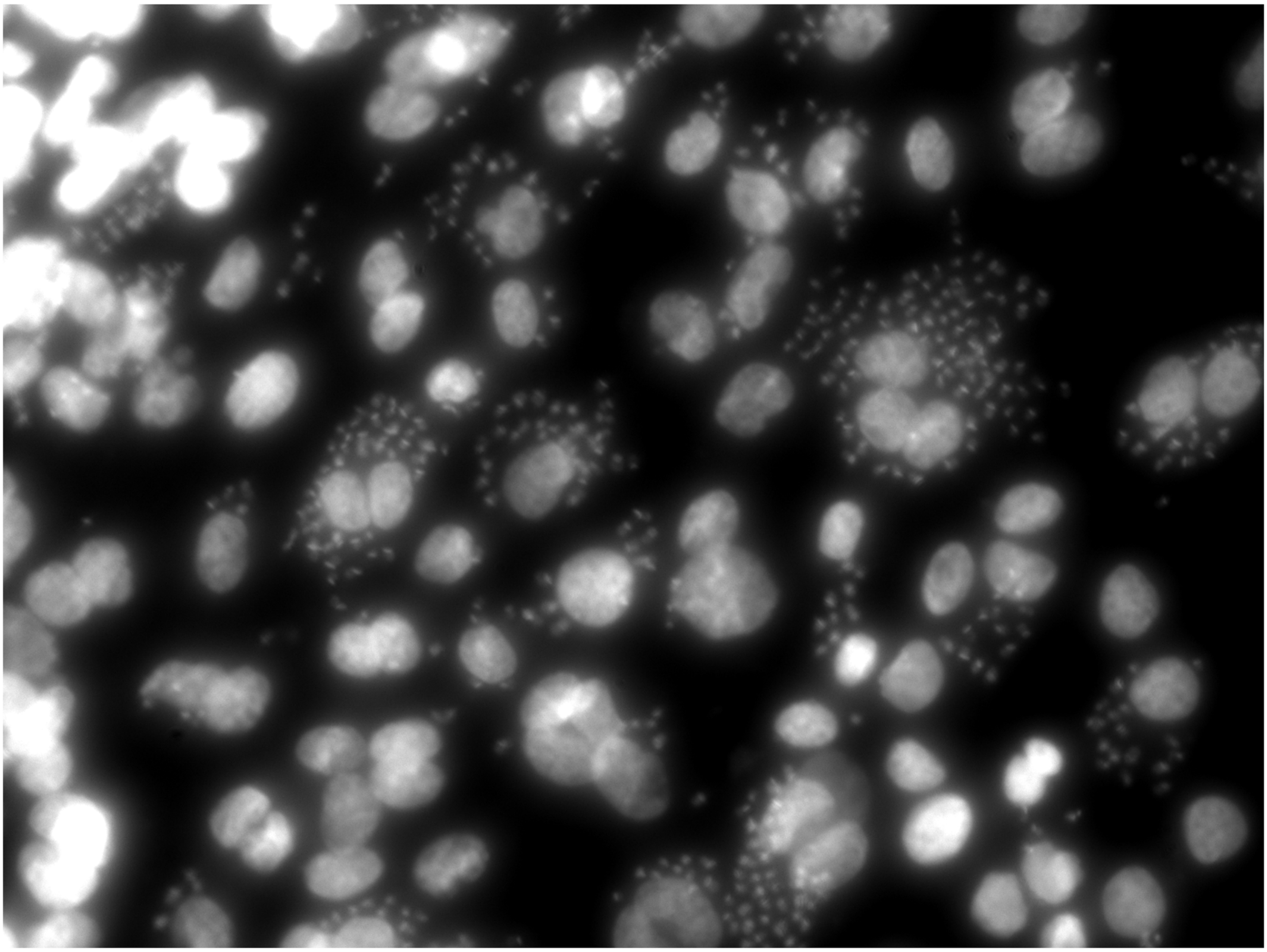

T. cruzi-infected hiPSC-CM

Fig 1H

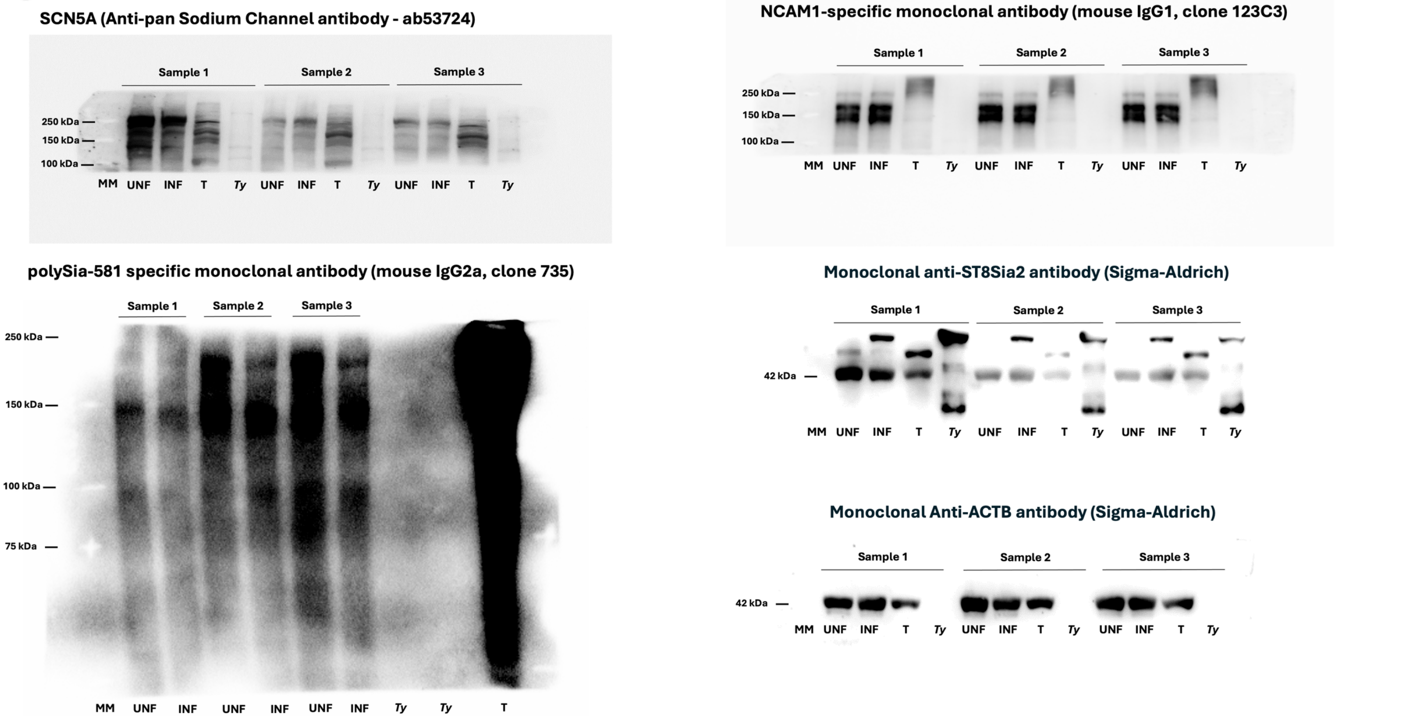

UNF – uninfected hiPSC-CM; INF – *T. cruzi*-infected hiPSC-CM; T - TE671 cell line; *Ty* – *T. cruzi* trypomastigote; MM – Molecular Marker.

S2C Fig

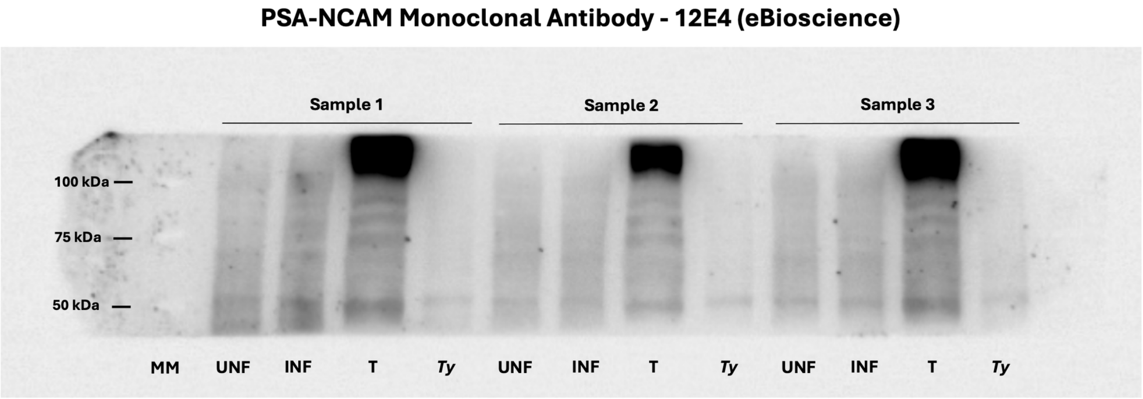

UNF – uninfected hiPSC-CM; INF – *T. cruzi*-infected hiPSC-CM; T - TE671 cell line; *Ty* – *T. cruzi* trypomastigote; MM – Molecular Marker.

Fig 2A

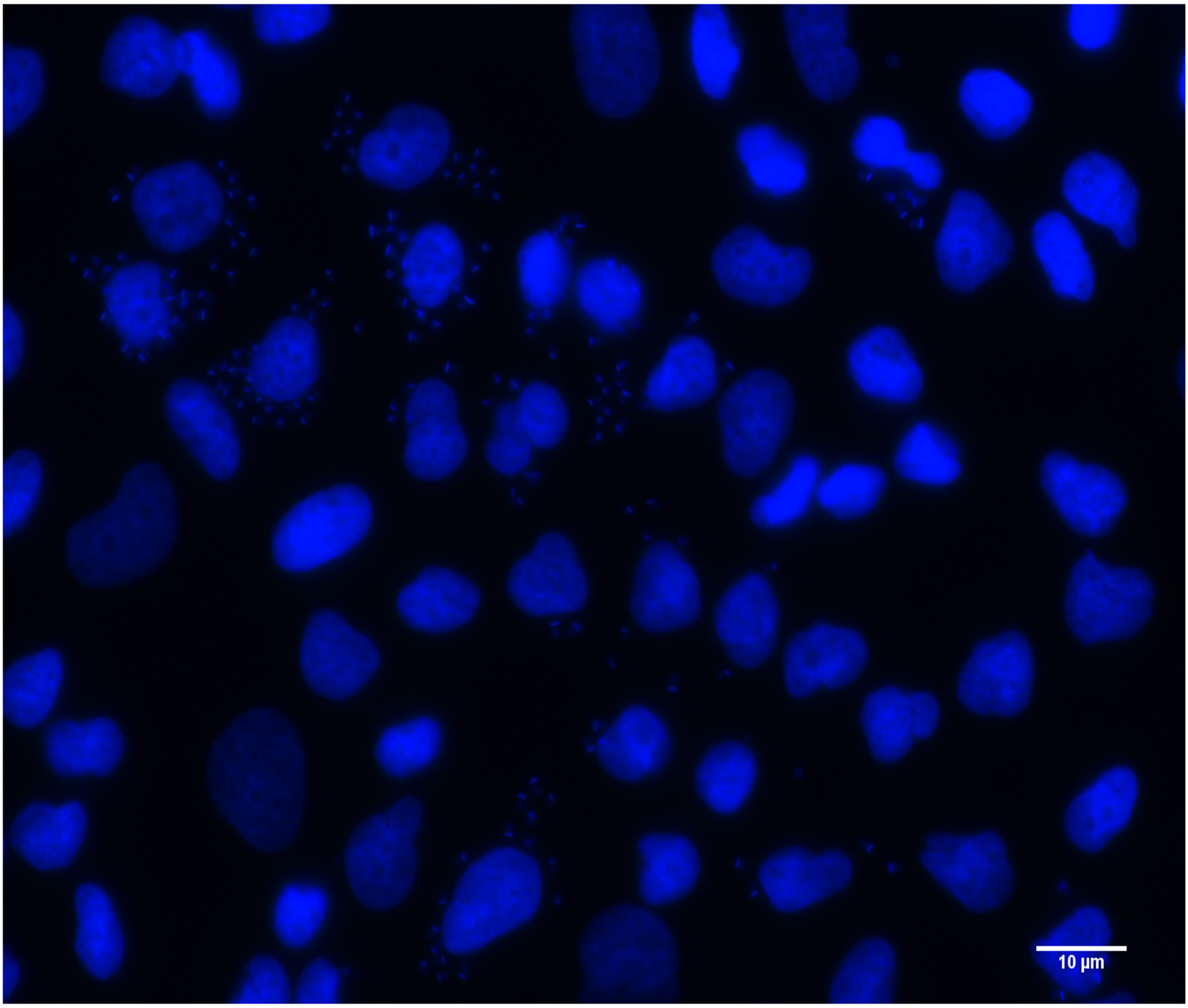

Fig 2H

Monoclonal anti-ST8Sia2 antibody (Sigma-Aldrich)

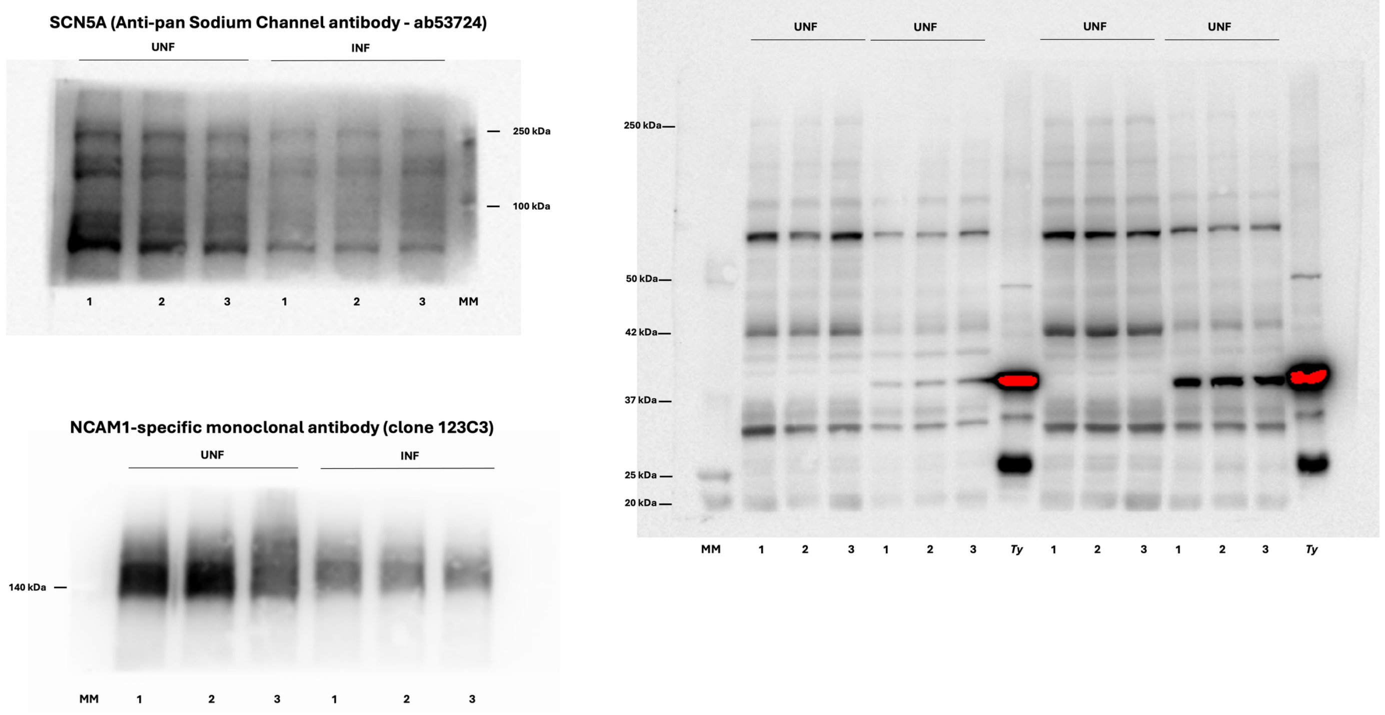

UNF – uninfected SH-SY5Y cells; INF – *T. cruzi*-infected SH-SY5Y cells; *Ty* – *T. cruzi* trypomastigote; MM – Molecular Marker

Fig 2H

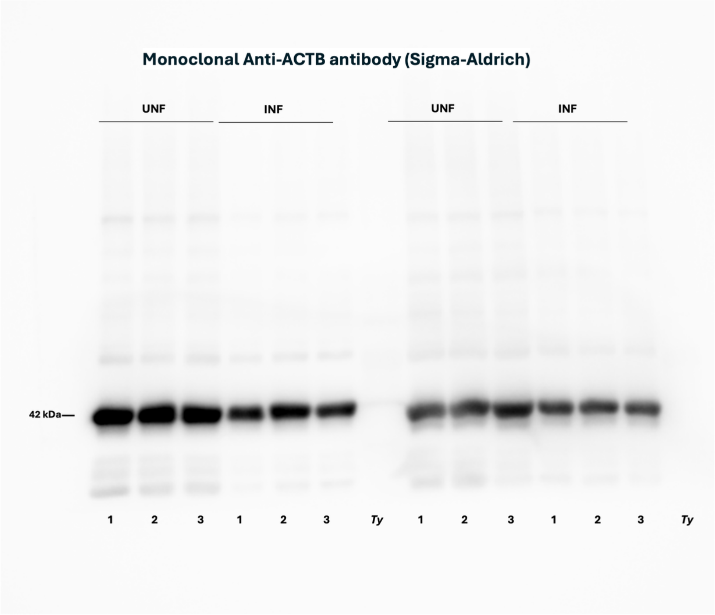

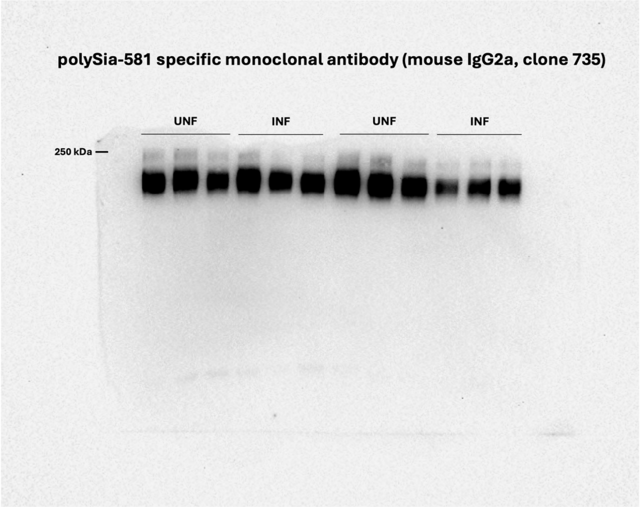

UNF – uninfected SH-SY5Y cells; INF – *T. cruzi*-infected SH-SY5Y cells; *Ty* – *T. cruzi* trypomastigote; MM – Molecular Marker

Fig 2H

PSA-NCAM Monoclonal Antibody - 12E4 (eBioscience)

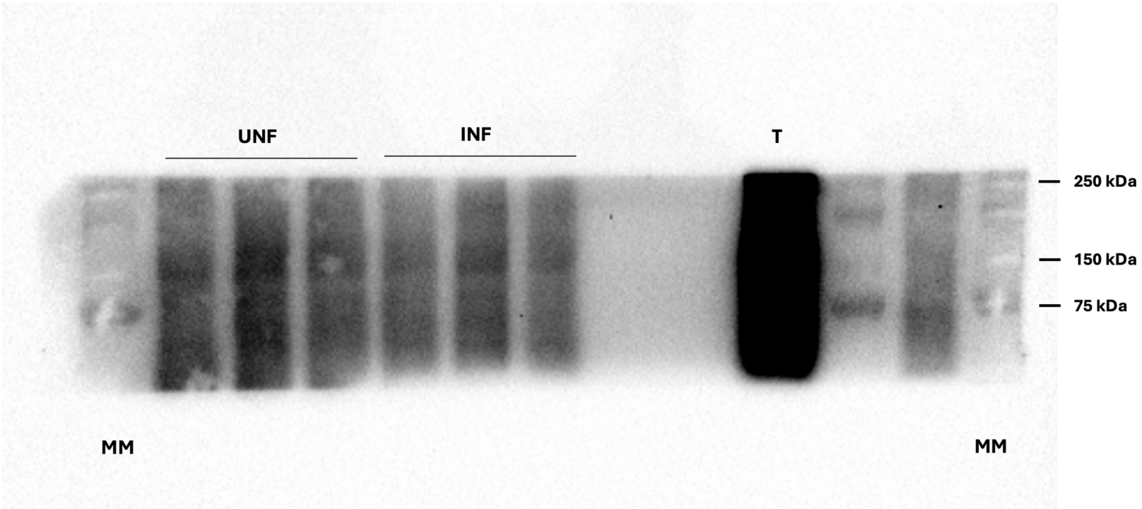

UNF – uninfected SH-SY5Y cells; INF – *T. cruzi*-infected SH-SY5Y cells; *Ty* – *T. cruzi* trypomastigote; MM – Molecular Marker

Fig 3A

Uninfected

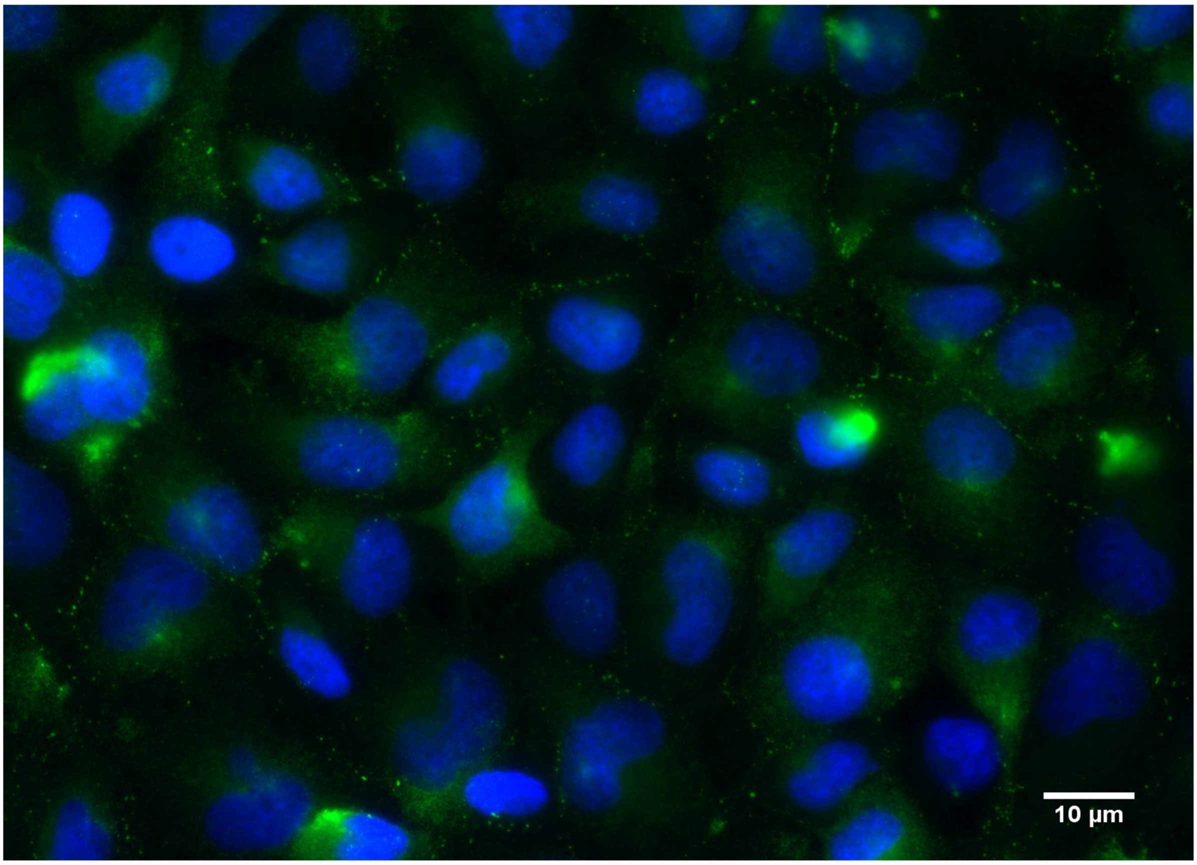

DAPI ST8Sia2

Fig 3A

Infected

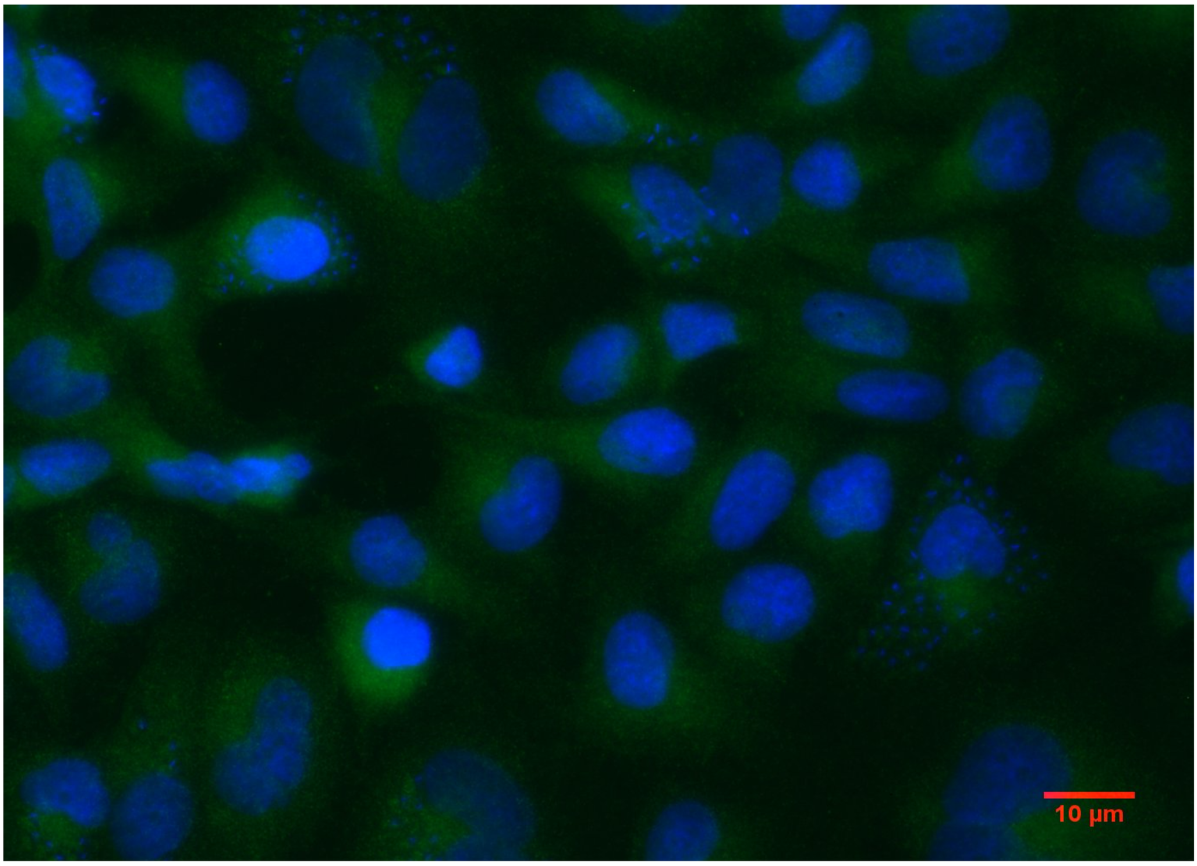

DAPI ST8Sia2

Fig 3B

Uninfected

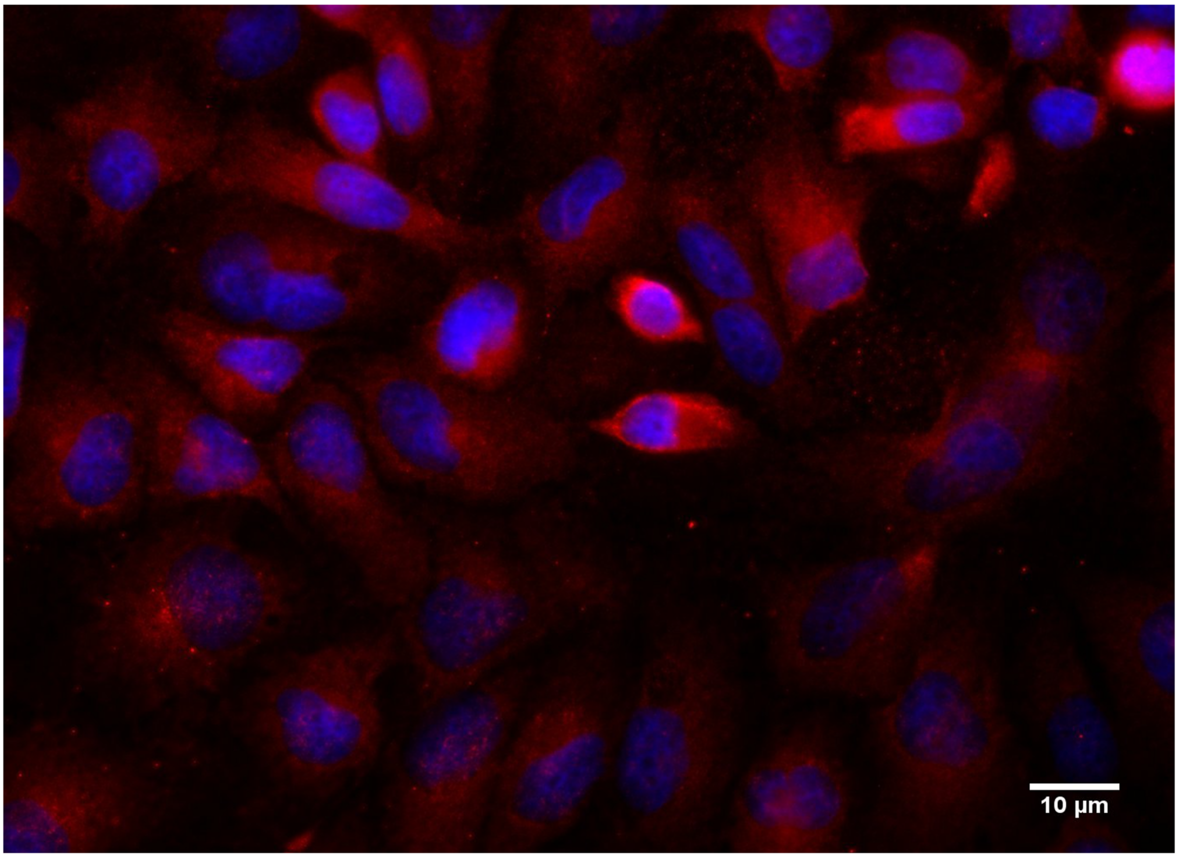

DAPI polySia

Fig 3B

Infected

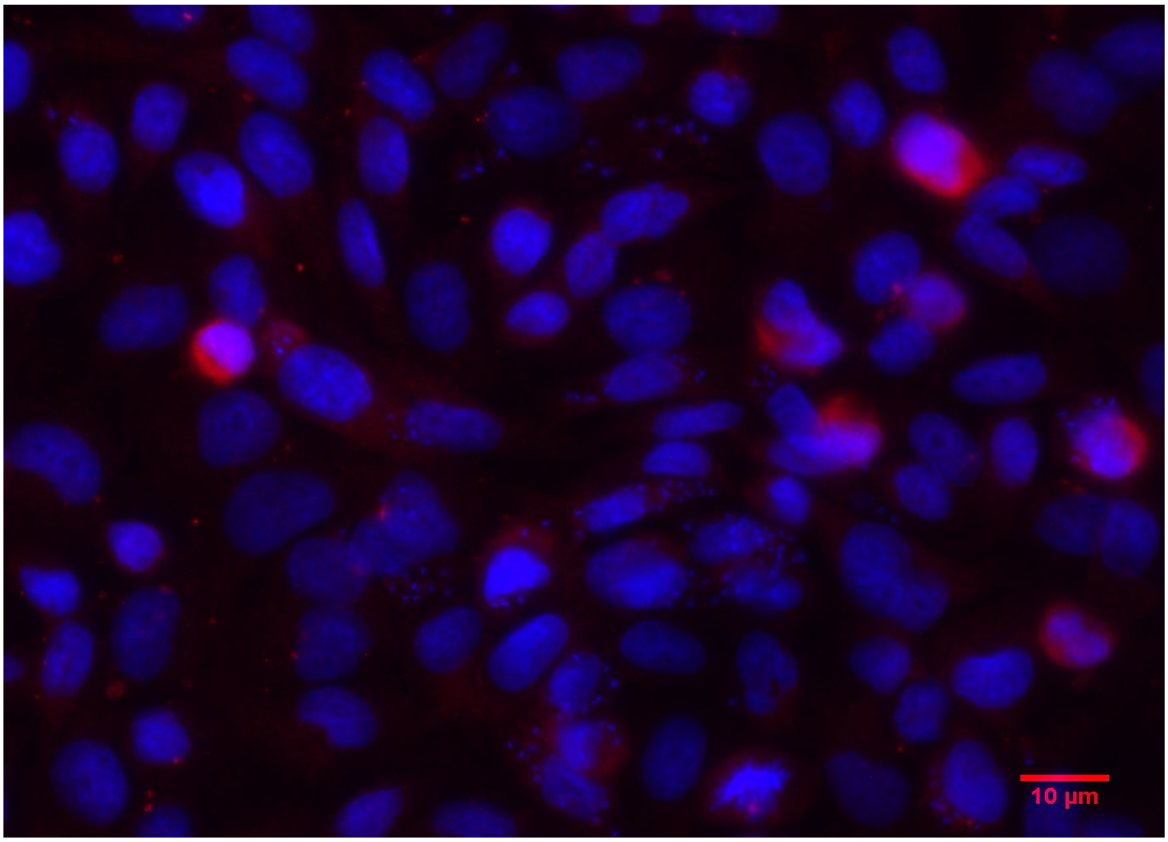

DAPI polySia

Fig 4C

Medium

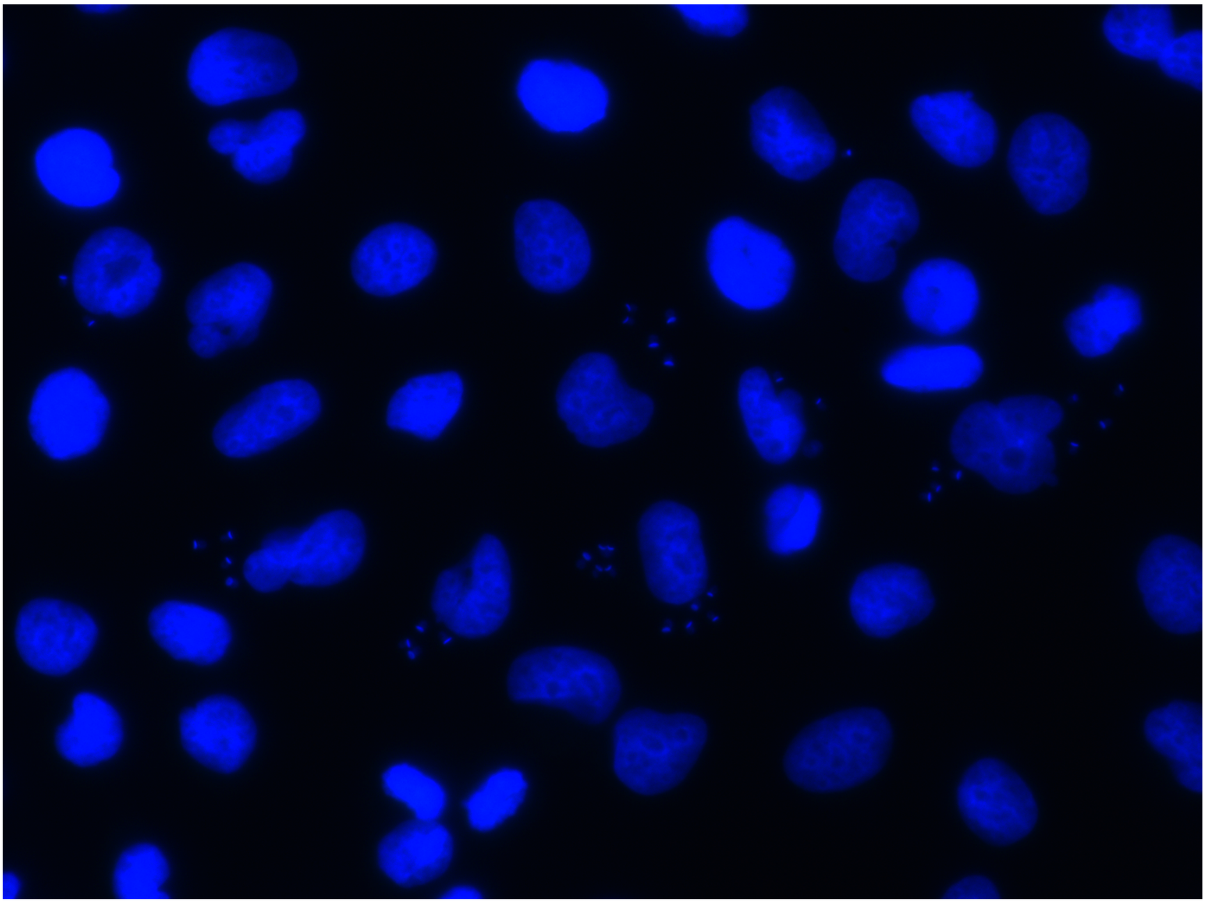

Fig 4C

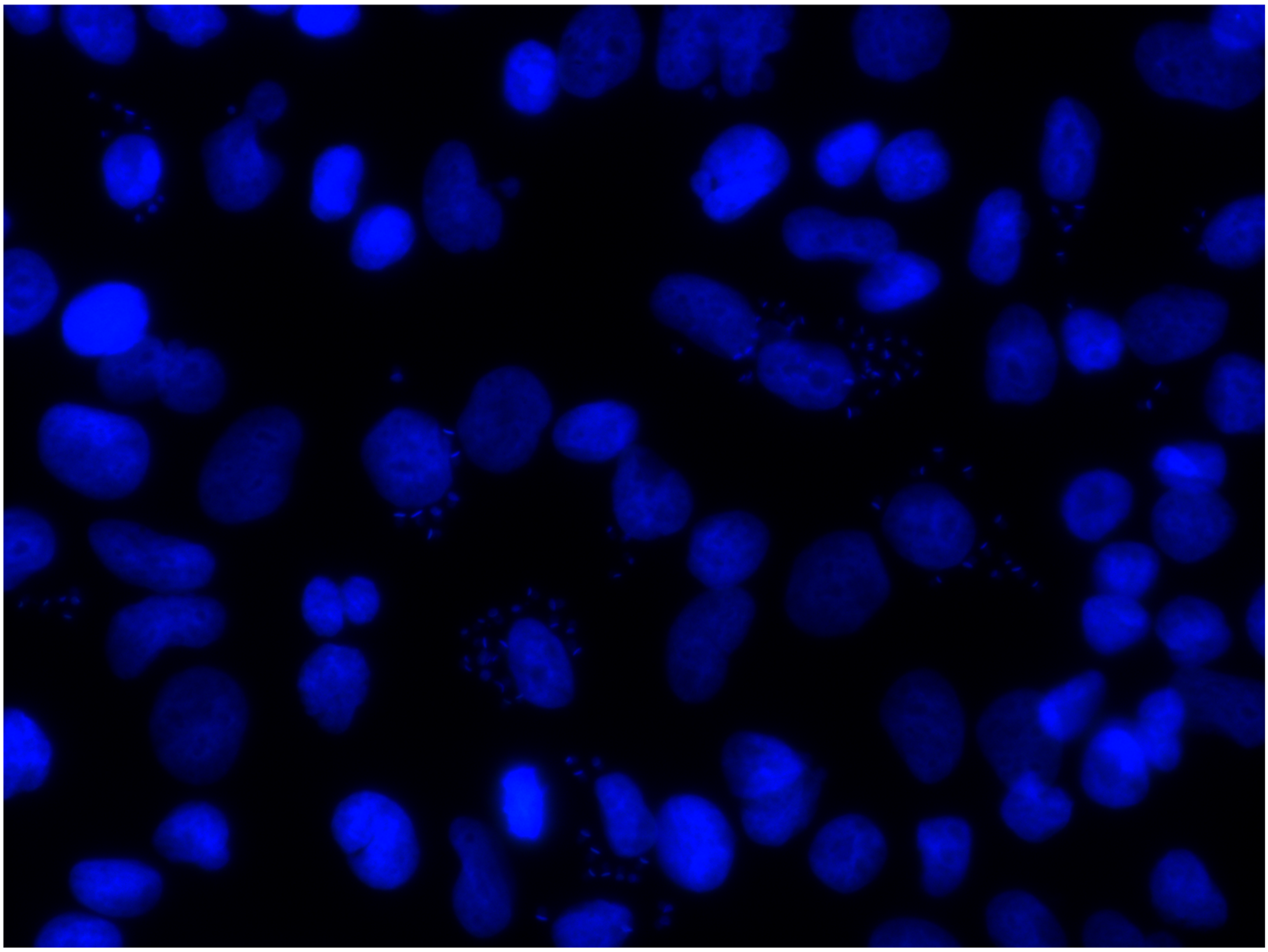

Endoneuraminidase-N (EndoN)

Fig 5F

Medium

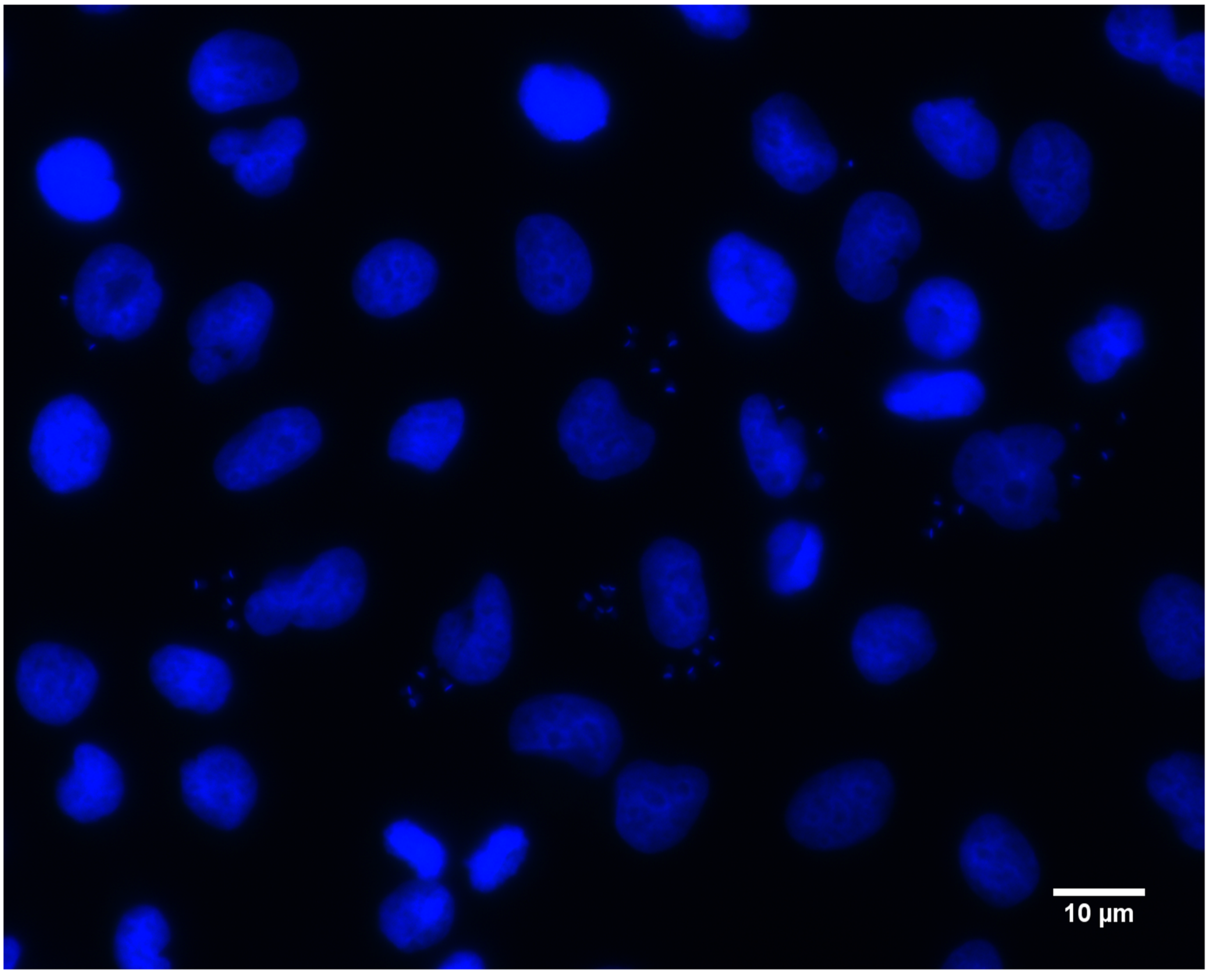

Fig 5F

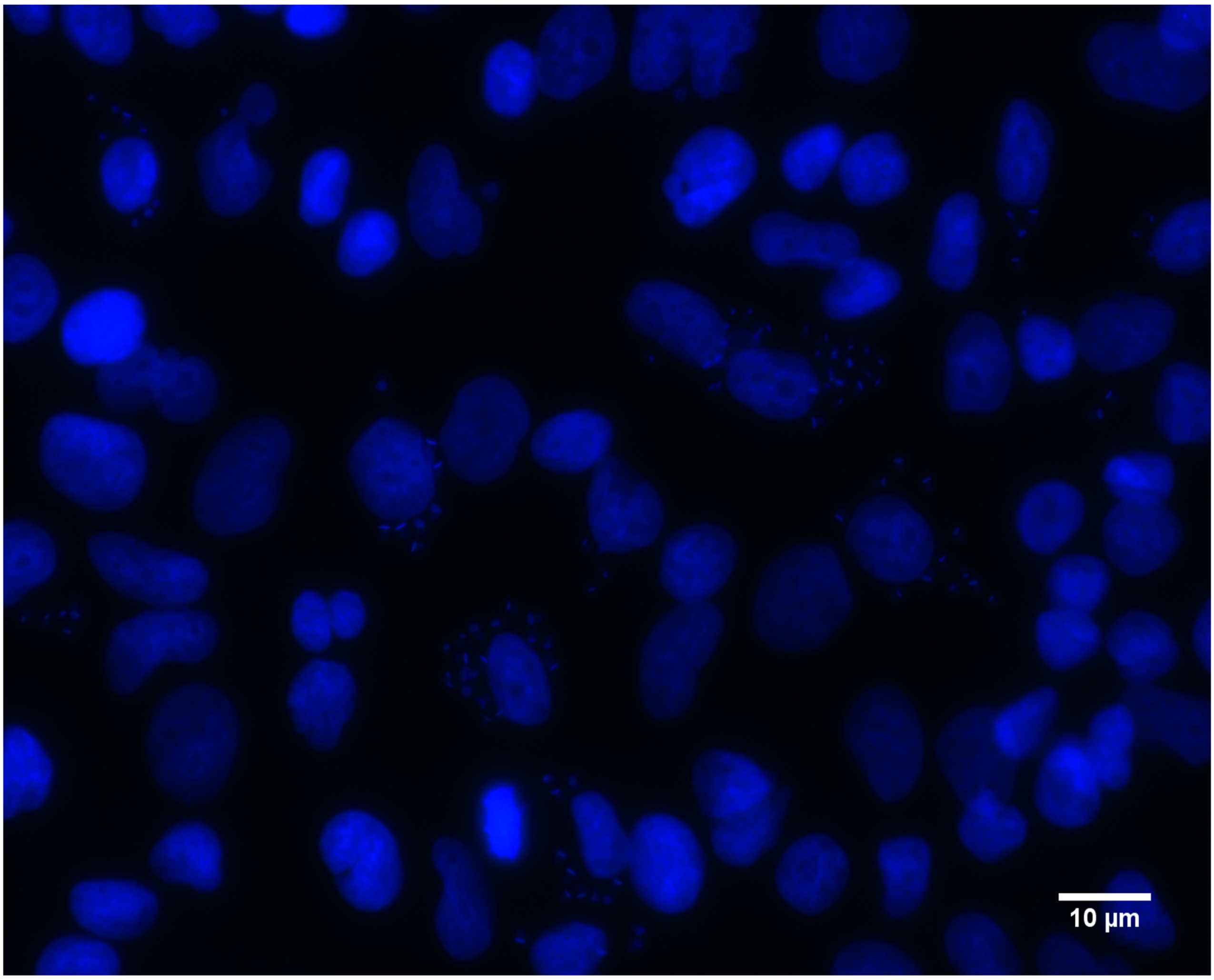

cytidine 5'-monophosphate (CMP)

Fig 5F

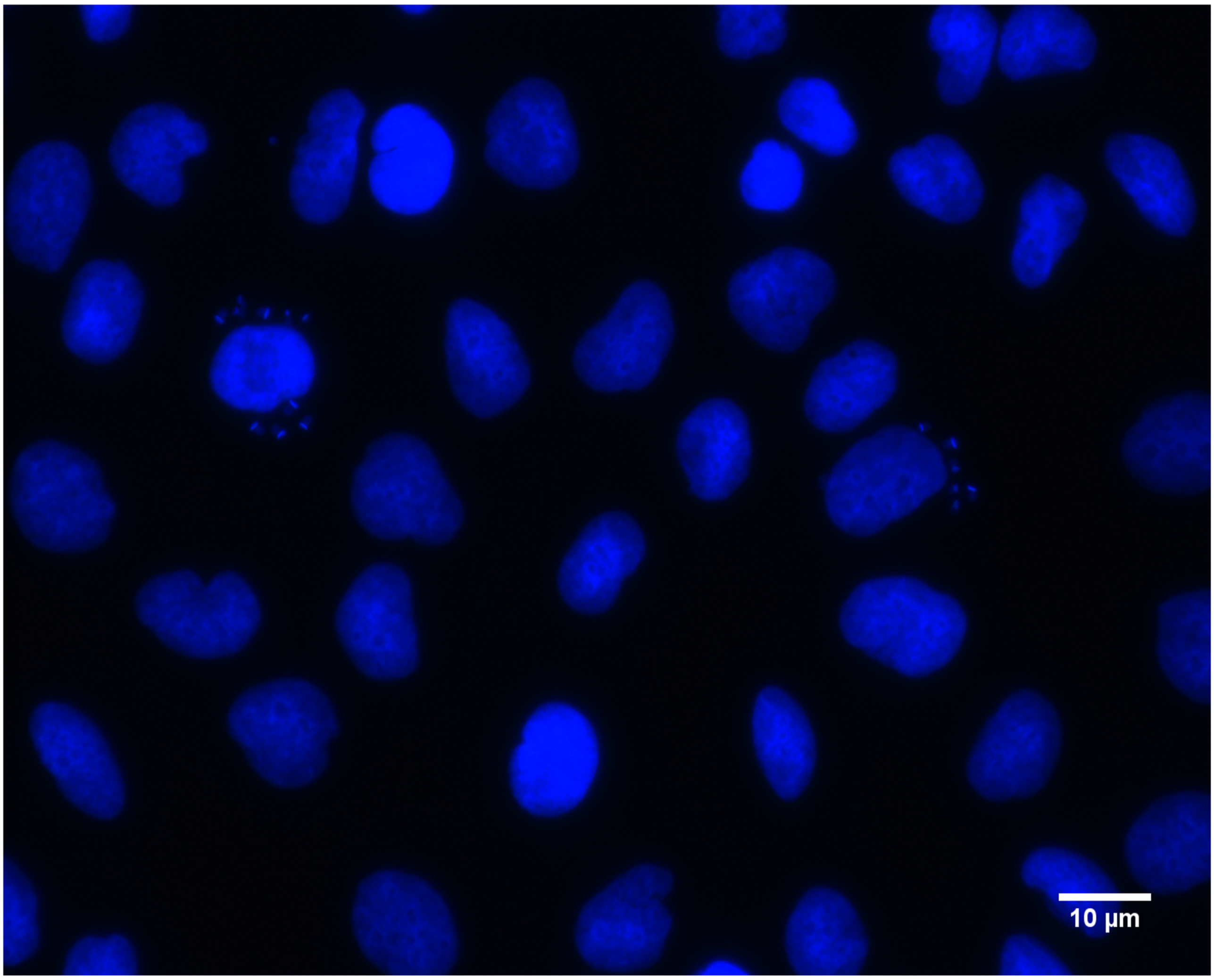

guanosine 5'-monophosphate (GMP)

Fig 5G

Medium

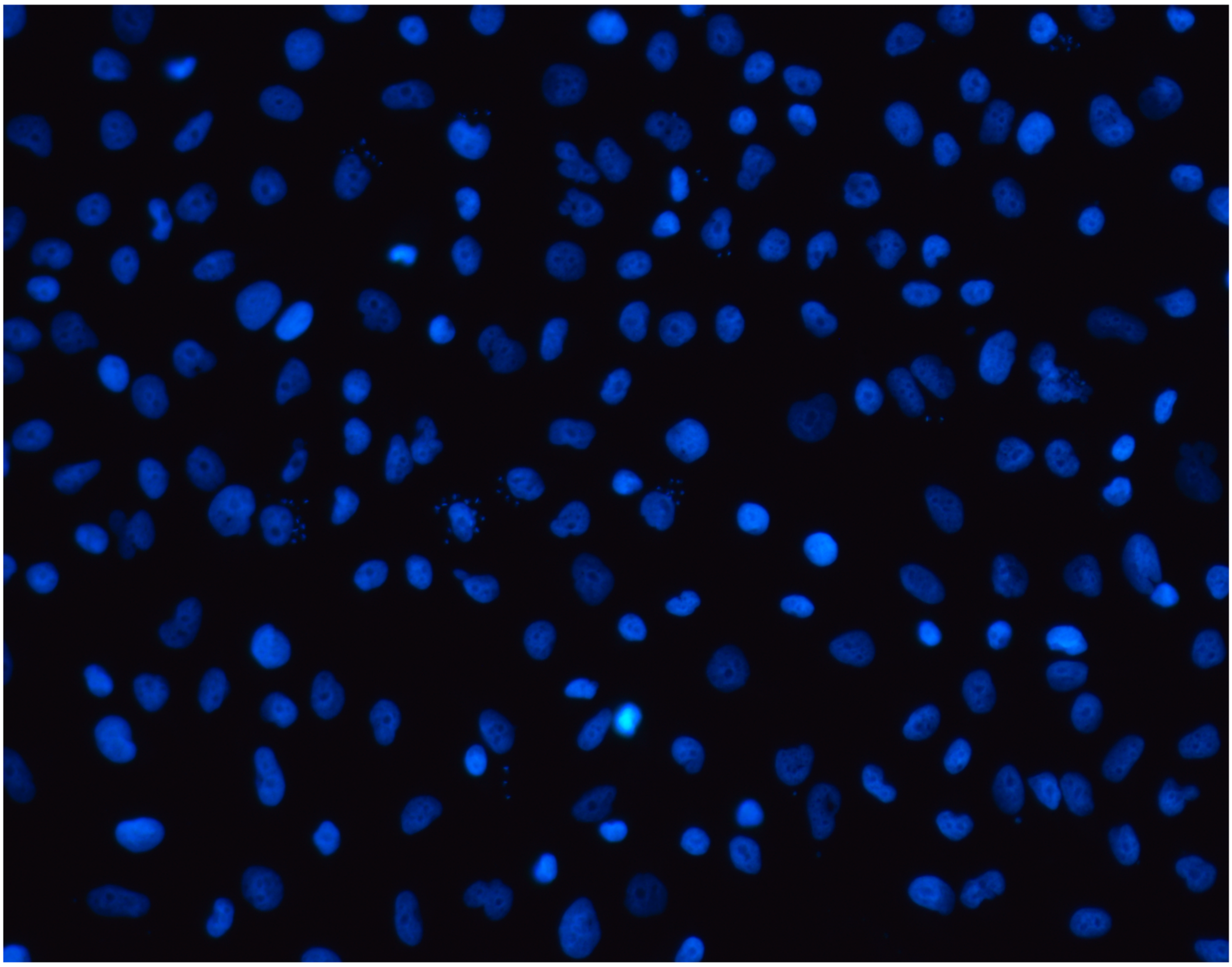

Fig 5G

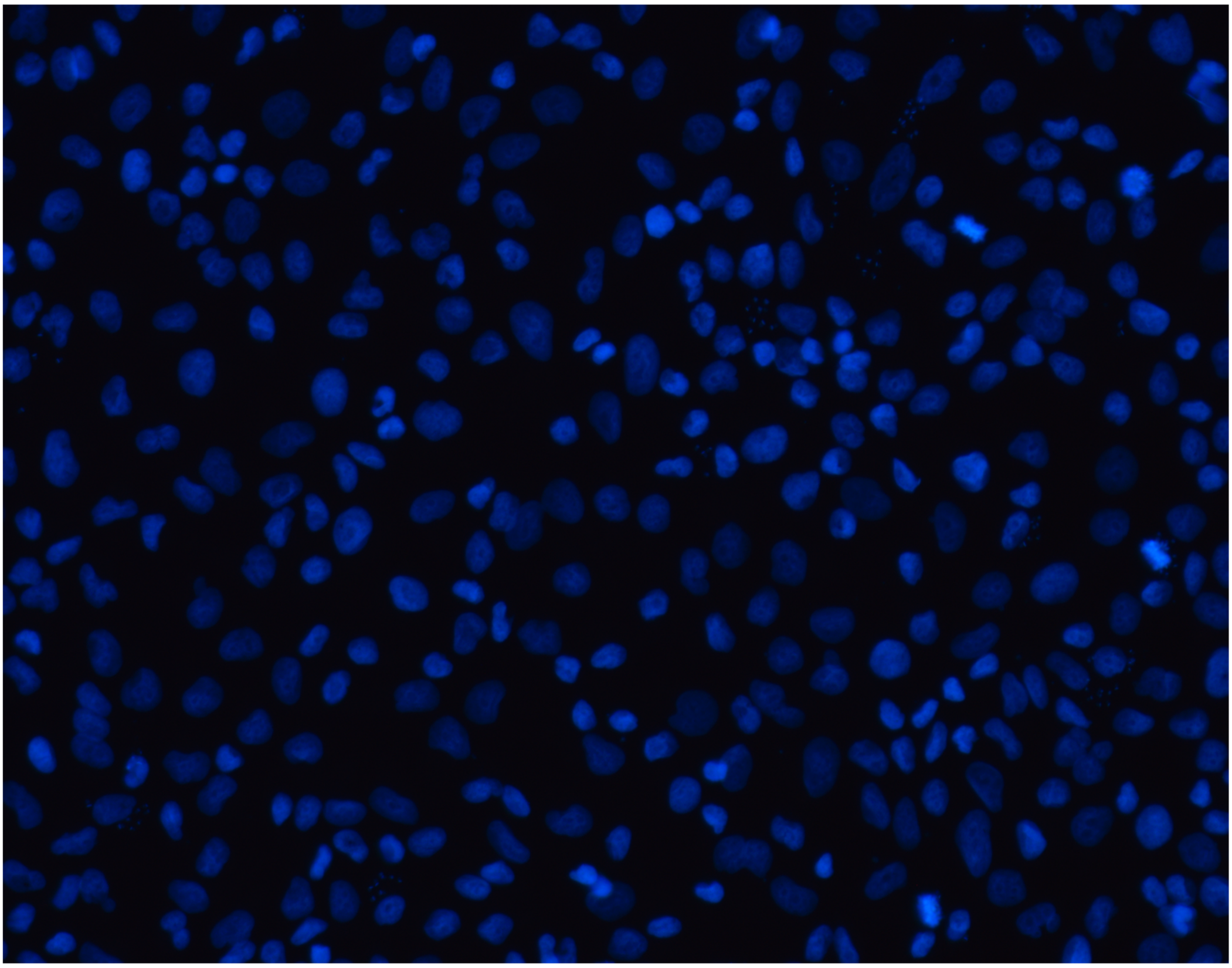

siRNA Negative Control (NTC)

Fig 5G

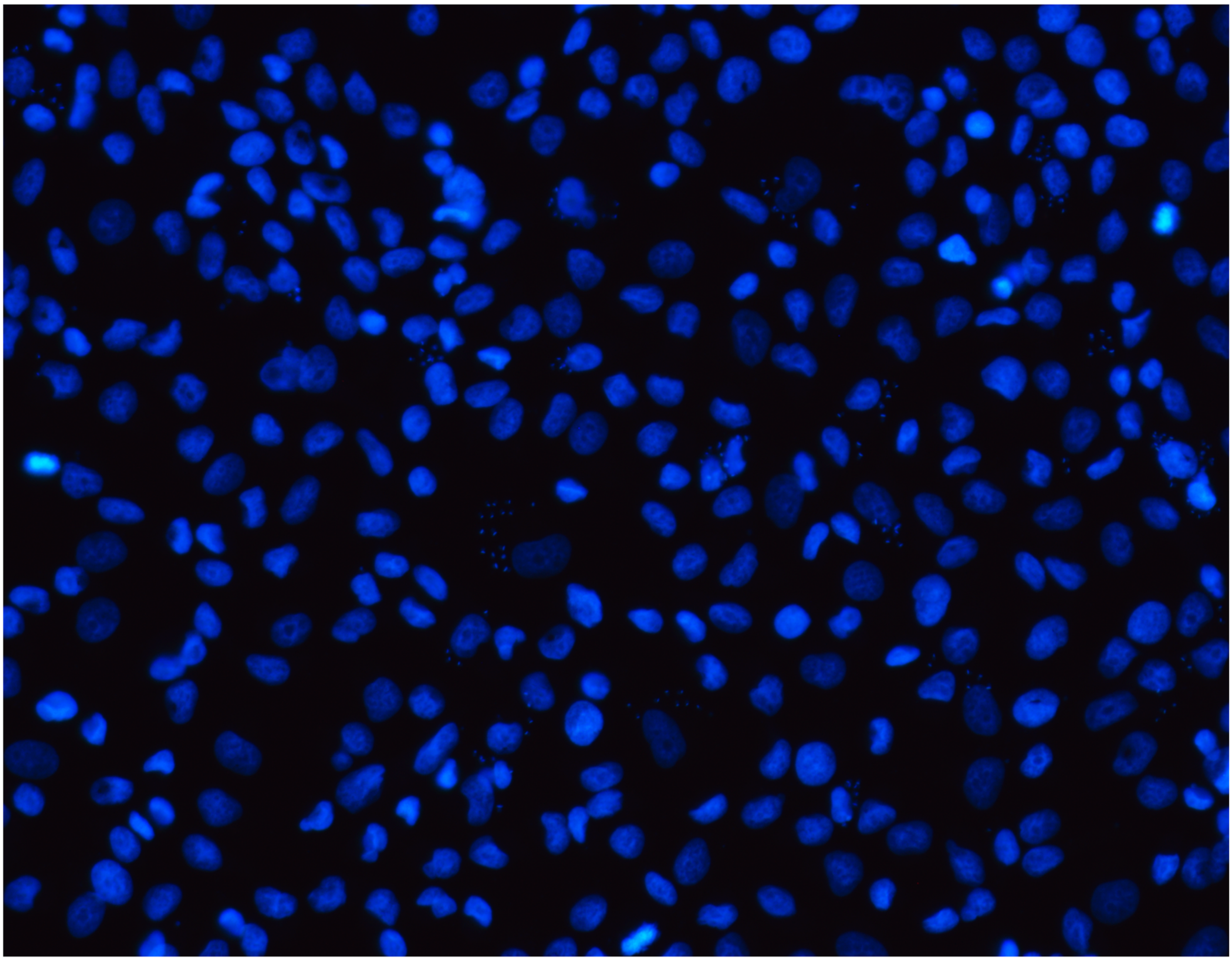

ST8SIA2 siRNA (siST8Sia2)

S3E Fig

Medium

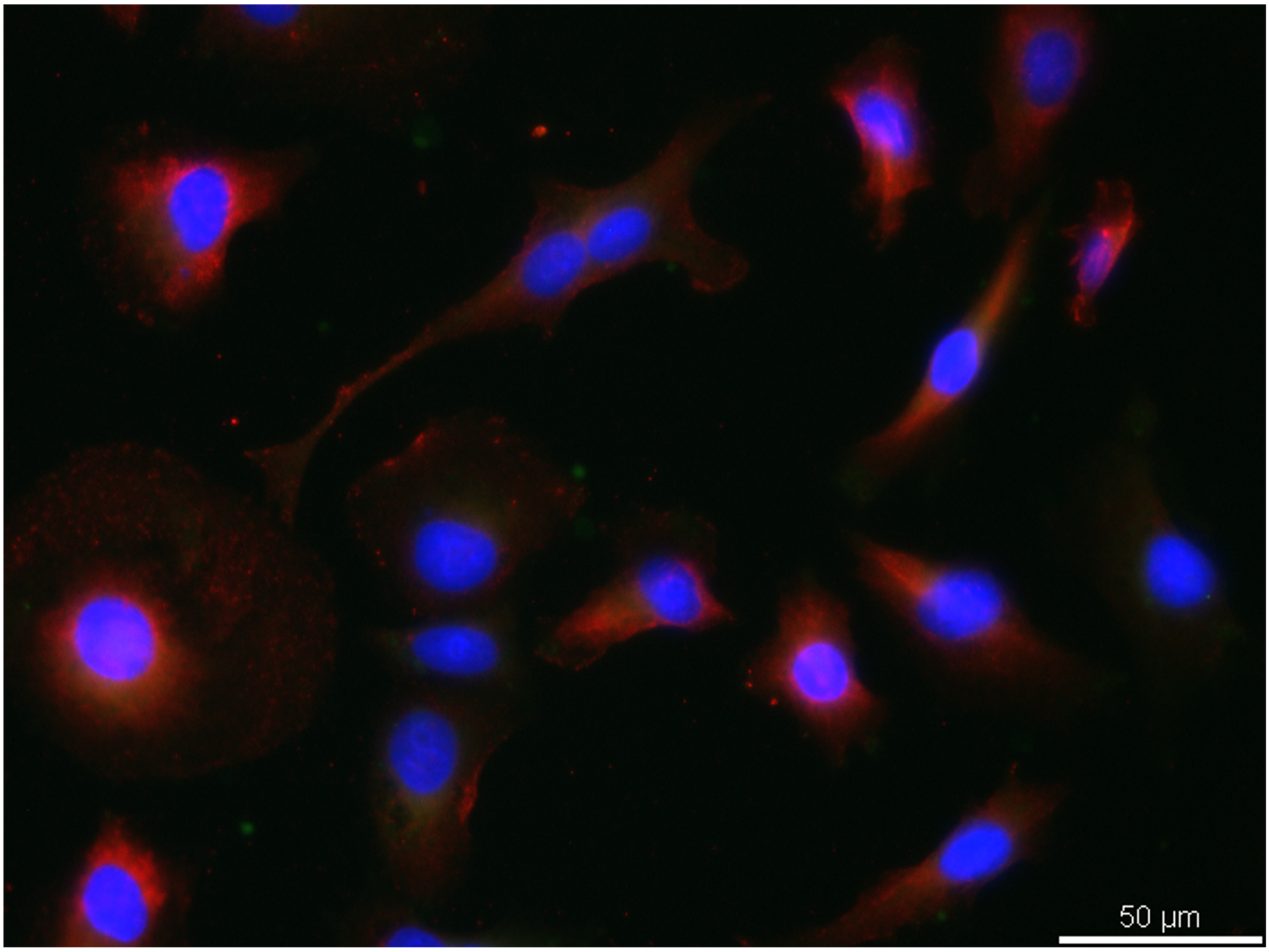

S3E Fig

siRNA Negative Control (NTC)

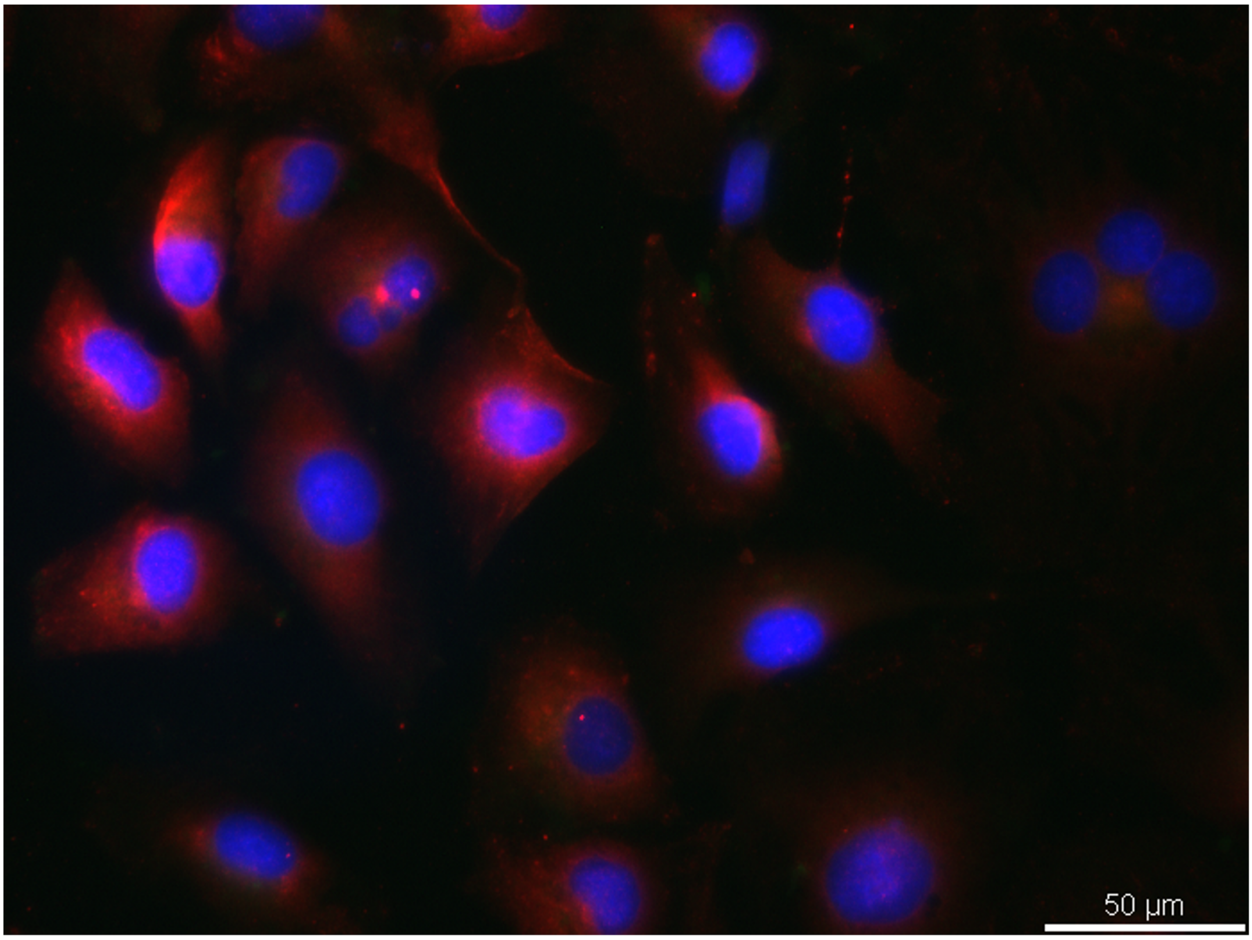

S3E Fig

ST8SIA2 siRNA (siST8Sia2)

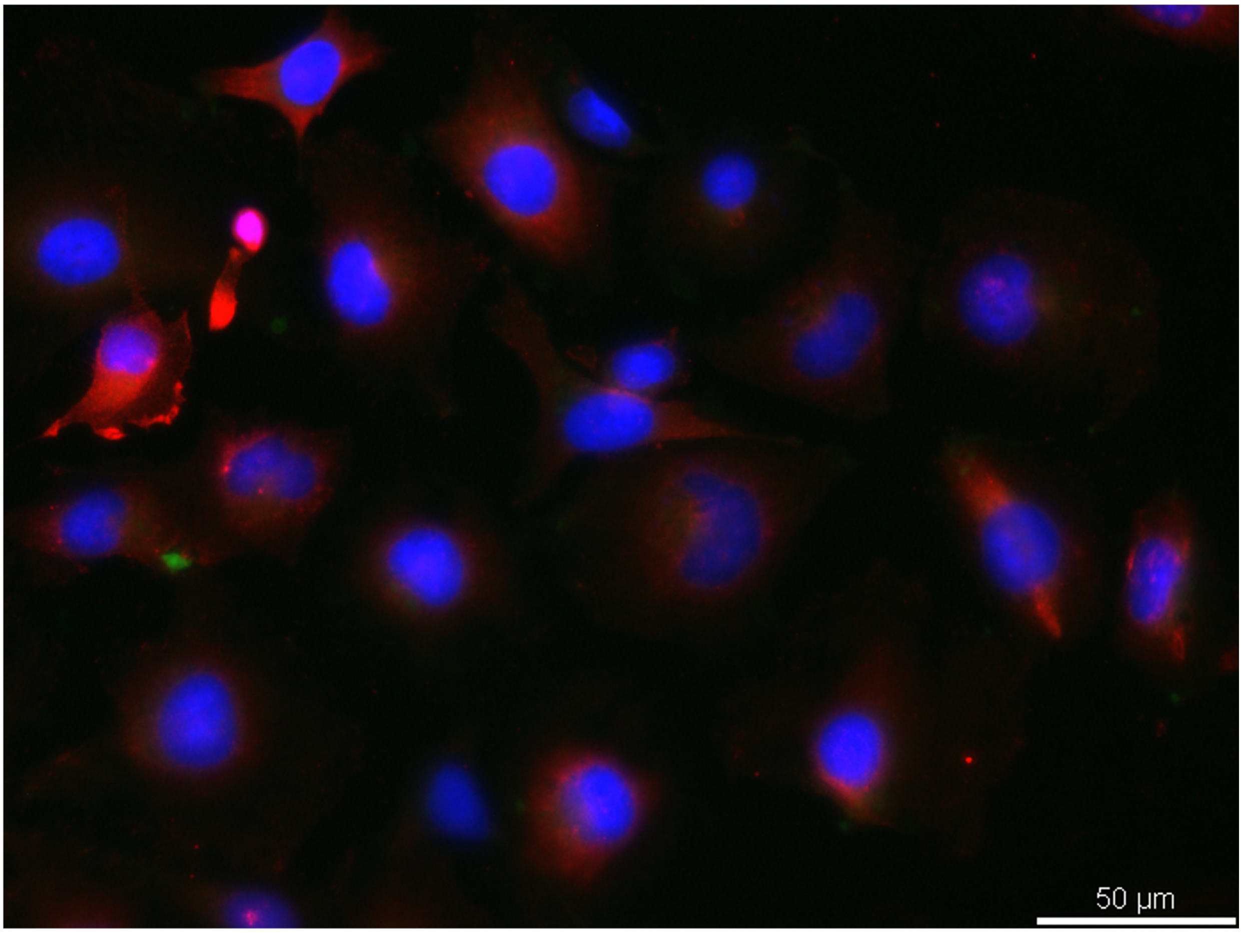

Representative chromatogram of the calibration curve used for quantification of polySia in *T. cruzi*-infected SH-SY5Y cells

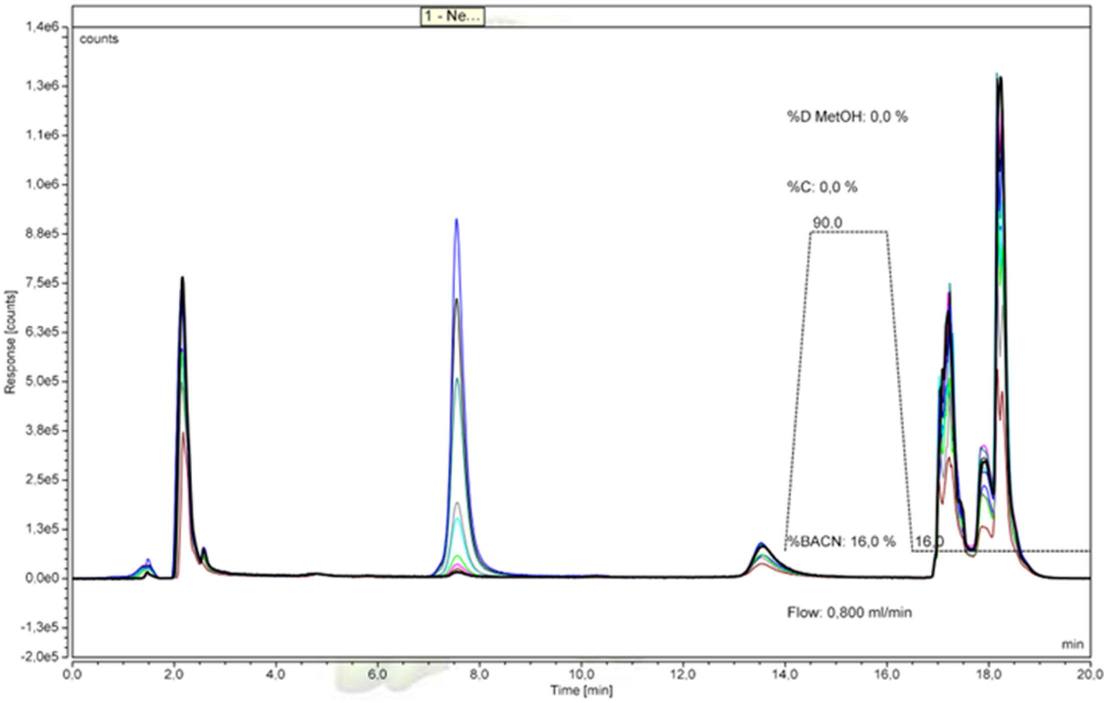

Chromeleon 7,
Version 7.2.10.23925, Thermo Fisher Scientific

Page 1 of 1

Printed by Vanquish
09/07/24 16:24

Representative chromatogram comparing the peaks of Neu5Ac obtained from *T. cruzi*-infected SH-SY5Y cells (blue) and uninfected SH-SY5Y cells (black), with the chromatogram for the injection of a blank shown in pink.

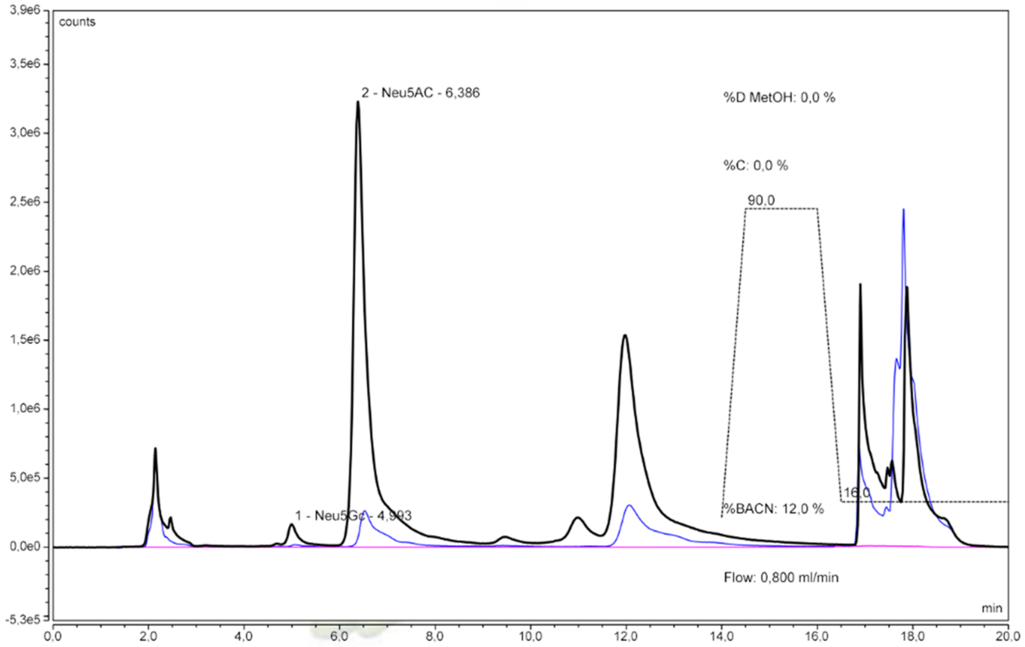

Chromeleon 7,
Version 7.2.10.23925, Thermo Fisher Scientific

Page 1 of 1

Printed by Vanquish
06/07/24 14:28

Representative chromatogram used for the quantification of polySia in SH-SY5Y treated with siRNA negative control (NTC).

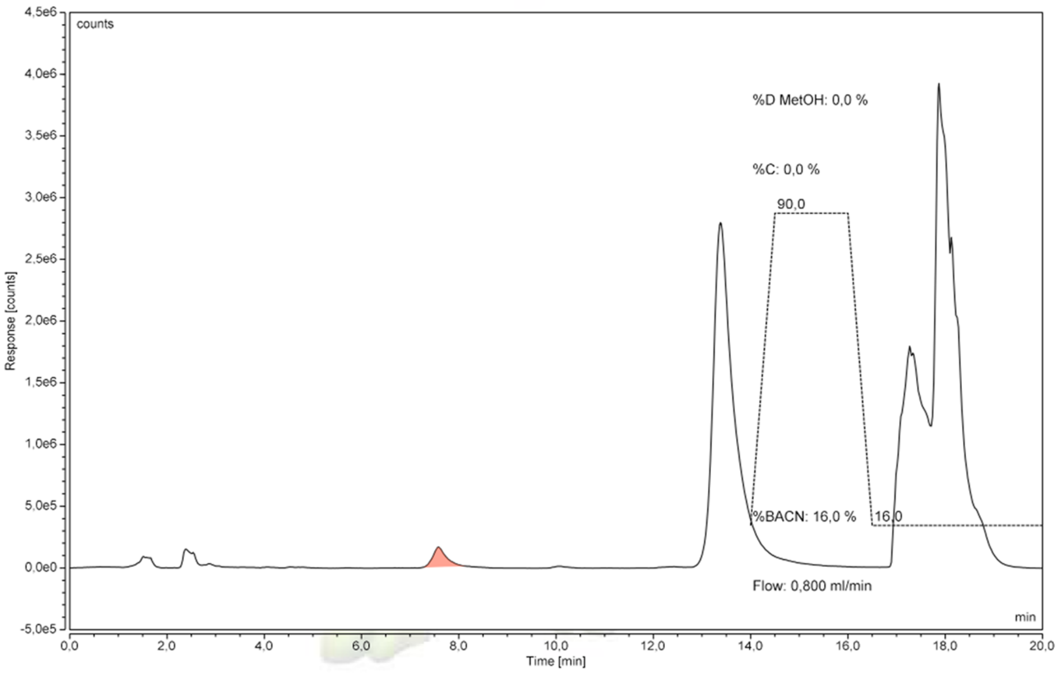

Representative chromatogram used for the quantification of polySia in SH-SY5Y cells treated with CMP, a pharmacological inhibitor of ST8Sia2

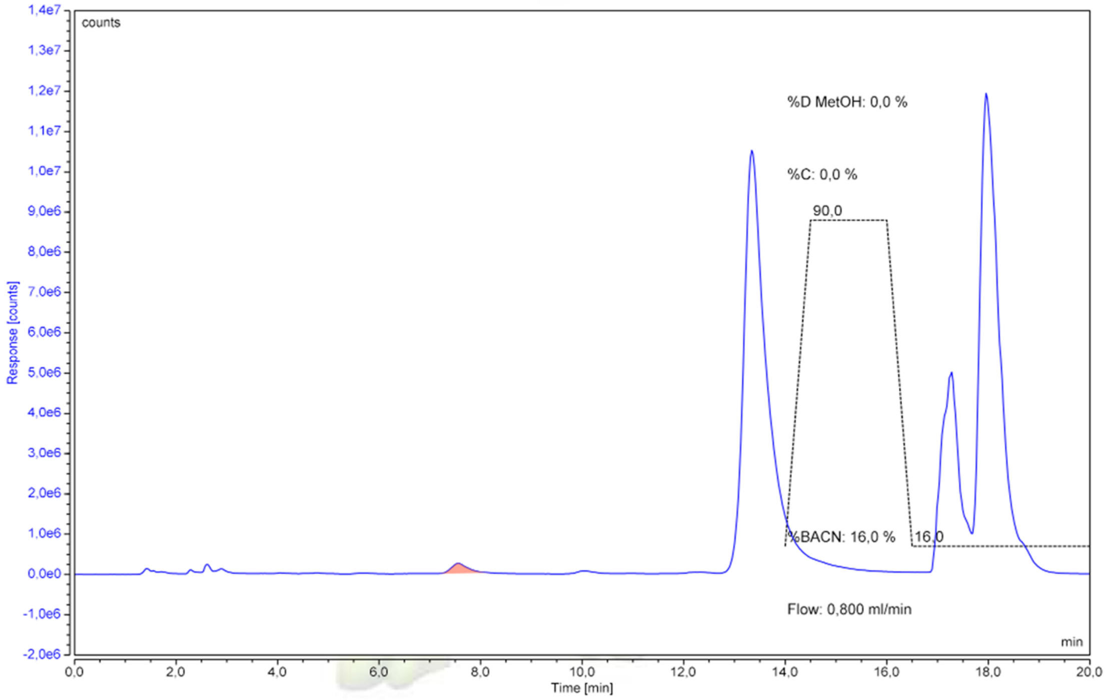

Representative chromatogram used for the quantification of polySia in SH-SY5Y cells treated with GMP, an analog molecule of CMP, without effect on the inhibition of ST8Sia2

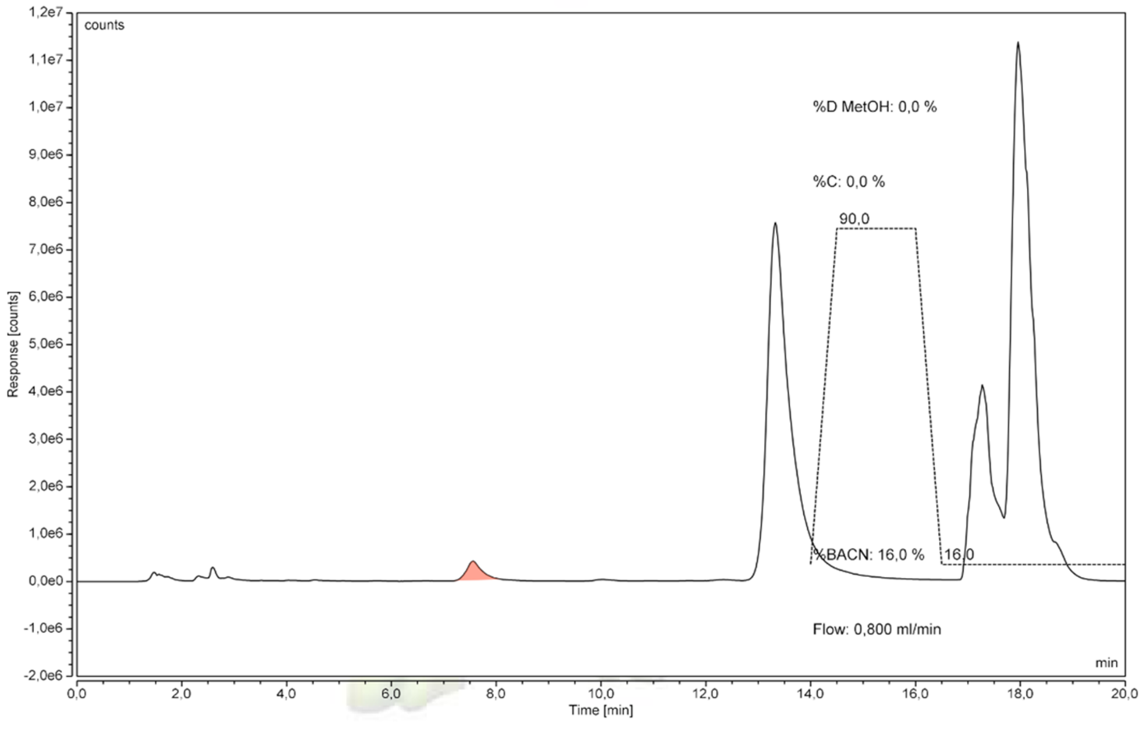

Representative chromatogram used for the quantification of polySia in SH-SY5Y cells with the *st8sia2* gene silenced (si*ST8Sia2*)

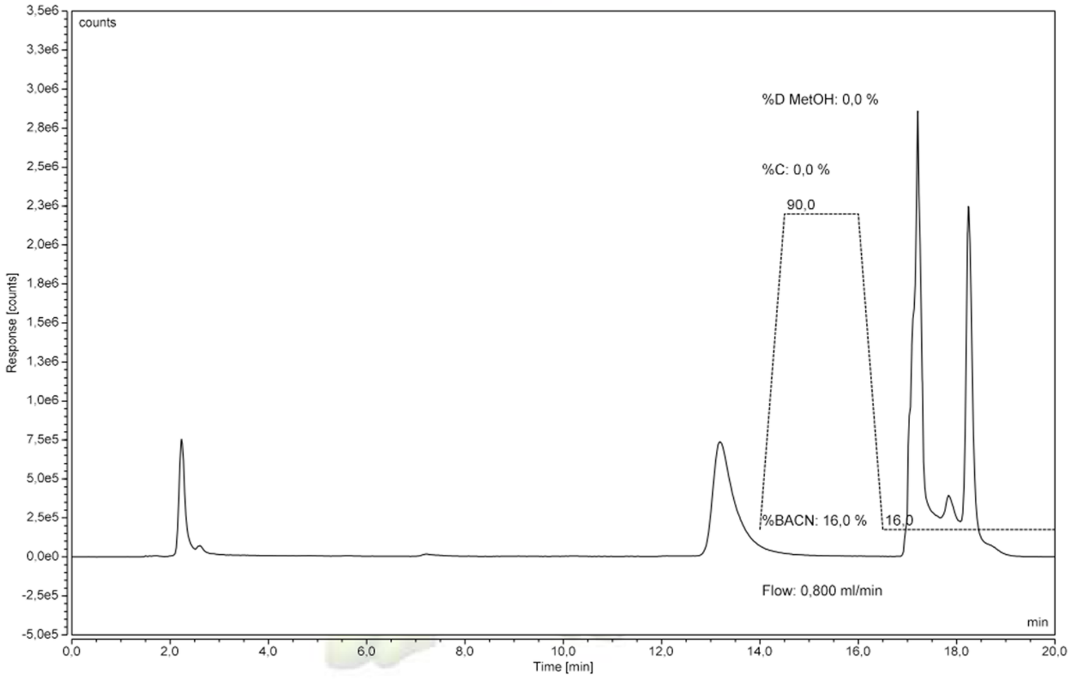

Supplement: S1 Data — (PDF) [file pntd.0012454.s007.pdf]
