# Supplementary material for: ST8Sia2 polysialyltransferase protects against infection by Trypanosoma cruzi
Source: PLoS Negl Trop Dis. 2024 Sep 25;18(9):e0012454. doi: 10.1371/journal.pntd.0012454 (PMC11466412; doi:10.1371/journal.pntd.0012454)
Supplement: Attachment — Submitted filename: Reviewer_comments_final version.pdf [file pntd.0012454.s009.pdf]

Dear Prof. Walderez Dutra,

Thanks for the reviewers' comments and suggestions that have helped improving the manuscript. We have revised the manuscript and included more data to support a potential mechanism of host ST8Sia2 modulation upon *T. cruzi* infection. Moreover, we have followed the reviewers' suggestions and improved the Figures as reported below. Thanks again to you and the reviewers for the time and commitment to help improving this manuscript.

Reviewer #1: *This is an interesting paper that proposes that polysialic acid (synthesised by ST8Sia2) inhibits host cell invasion (and/or propagation?) by T. cruzi and that this is why T. cruzi infection of cells reduces ST8Sia2 expression. The data presented are well described but there are some missing controls, and the paper would benefit from more critical assessment of significance.*

The authors should address the following points:

1. While the phenomena described are certainly noteworthy, it is difficult to understand an overall hypothesis as to why the phenomenon of T. cruzi infection of host cells causing downregulation of ST8Sia2 would influence an in vivo infection. Thus, the downregulation of ST8Sia2 in an individual cell would be post-invasion and unlikely(?) to influence cell invasion by more parasites, especially as it would take many hours for pre-existing polysialic acid to be turned over by the invaded host cell. The authors should help the reader understand how they think the T. cruzi invasion induced downregulation of ST8Sia2 expression could assist host infection and parasite survival.

Reply: We thank Reviewer #1 for the comments and suggestions that have helped in improving the current version of the manuscript. We agree with the reviewer that the *ST8Sia2* downregulation is unlikely affecting cell invasion because *i)* we see changes in RNA levels only starting at 24 and 48 hours based on the transcriptomic data and on qRT-PCR data. This time point is associated with the intracellular stages of the parasite; *ii)* The *in vitro* infection with *T. cruzi* involves an initial time of 3 hours for adhesion, then the parasite that did not adhere and entered the cells are washed out before continuing the experiment. Due to that, we believe that at 24 and 48 h.p.i, we have almost no parasites in the extracellular media; *iii)* According to Al-Saraireh *et al.* (doi: 10.1371/journal.pone.0073366), the polysialic acid turnover is 6 hours which will be a late time point for the invasion.

The analysis of the *T. cruzi* infected *vs* uninfected cells showed a lower level of ST8Sia2 which correlates with lower levels of polysialic acids. We have detected lower levels of ST8Sia2 substrates such as SCN5A in human cardiomyocytes and NCAM and SCN5A in SH-SY5Y. Previous reports have shown that deletion of ST8Sia2 in mice induce cardiac alterations (doi: 10.1073/pnas.0905414106; doi: doi: 10.1242/dev.199431). We have identified lower levels of ST8Sia2 in infected cells (cardiomyocytes) compared to uninfected. One hypothesis on the effect of polySia with the *Trypanosoma cruzi* cell survival could be related to the disbalance of oxidative stress.

It is known that *T. cruzi* infection needs oxidative signals to infect macrophages (https://doi.org/10.1371/journal.pntd.0004555). Moreover, it is known that oxidative stress and DNA damage are activated during *T. cruzi* infection of HeLa cells (10.1371/journal.ppat.1009502). These results show the importance of ROS during the *T. cruzi* infection. It has been reported that sialic acid deficiency is associated with oxidative stress (10.1093/hmg/ddx192). This was observed in GNE-related mutations leading to muscle atrophy

and weakness. In this article, it is observed that lower levels of sialic acid increase the oxidative stress, seen by increase protein S-nitrosylation. It has been shown that hypoxia leads to oxidative stress (https://doi.org/10.1016/j.freeradbiomed.2018.03.042), and hypoxia leads to a reduction in polySia expression (https://doi.org/10.1038/srep33026). Due to that, the data obtained in this study indicate that lower levels of ST8Sia2 reduces polySia which in turn increases the oxidative stress favouring *T. cruzi* survival and proliferation. Interestingly, it has been shown that host cells treated with glutathione precursor and N-acetyl cysteine reduce parasite burst significantly (10.1371/journal.ppat.1009502).

It has been shown in fluid shear stress in human endothelial cells, that sialic acids (Sia) removal alters kinase signaling involved in the nuclear accumulation of Nrf2 and promotes mitochondrial reactive oxygen species accumulation (10.1016/j.redox.2020.101816). Notably, knockdown of the endogenous sialidase NEU1 increases Nrf2 target gene expression such as HO-1, directly implicating sialic acid in the regulation of Nrf2 signaling (10.1016/j.redox.2020.101816). It has been shown that NEU1 cleaves polySia (10.1371/journal.pone.0146398). Due to that, higher levels of NEU1 could reduce polySia levels, increase oxidative stress and so on parasite survival. It has been shown that HO-1 overexpression reduces *T. cruzi* parasitism *in vitro* and *in vivo* (https://doi.org/10.1371/journal.ppat.1008599). Due to that, a reduction obtained by modulation of NRF2 accumulation due to low levels of polySia will increase the *T. cruzi* infection.

To confirm this hypothesis, we silenced *ST8Sia2* and monitored the modulation of oxidative stress response in SH-SY5Y *ST8Sia2* silenced (si*ST8Sia2*) that were: i) treated with antimycin A and N-acetylcysteine, activator and inhibitor of the generation of oxidative response, respectively; ii) infected with *T. cruzi*. Measuring the cytosolic levels of ROS, we detect difference (reduction) in contents of cytosolic ROS produced by si*ST8Sia2* cells. On the other hands, when we are measuring the cytosolic levels of ROS after the addition of antimycin A, we detected an increase in the cytosolic ROS produced. At the same time, we did not observe any difference in the cytosolic levels of ROS in the si*ST8Sia2* cells upon treatment with N-acetyl cysteine. In similarity, we also detected significantly changes in the levels of mitochondrial ROS when measured in the basal conditions (si*ST8Sia2 vs. siRNA control [NTC]* cells). However, we observed higher ROS levels in the mitochondria of cells with *ST8Sia2* silencing upon treatment with antimycin A, an inhibitor of oxidative phosphorylation. This effect was significant compared to the cells treated with siRNA control (NTC), cultivated as same conditions experimental. Treatment with N-acetyl cysteine, an antioxidant agent, revealed no changes in the *ST8Sia2* silenced cells. Notably, we also observed a higher level of calcium mitochondrial and disrupt in the mitochondrial membrane potential in the *ST8Sia2* silenced cells, suggesting that the modulation of *ST8Sia2* leads a modulation of pathways associated with stress responses (These findings are presented in Figure 7, new version of this manuscript).

Additionally, we used proteomics approach, and showed that the *ST8Sia2* silenced cells are more responsive to activation of oxidative stress pathways when infected by *T. cruzi*. Since, we identified proteins (showed in the figure 7, new version of this manuscript) associated with these pathways. Due to that, our data show that low levels of ST8Sia2 increase the susceptibility of SH-SY5Y cells to induce a high production of ROS and mitochondrial calcium. Then, higher ROS levels might create an environment favourable for *T. cruzi* replication.

2. While the data presented using immunofluorescent microscopy data appear sound, they are difficult to assess. For example, in Fig 1A what % of host cells are infected and with what number (average and SD and median) of amastigotes?

Reply: We thank Reviewer #1 for the suggestion. This figure has been modified in the new version of the manuscript (Figure 1B, new version of this manuscript). We have counted the number of amastigotes per cell and calculated the % of infected host cells.

3. The reduction in ST8Sia2 mRNA is clear (Fig 1B) but the reduction in ST8Sia2 protein is slight (Fig 1B) (presumably because it has a long half-life – are there any data in the literature from global turnover studies?) bringing into question the reduction in polysialic acid as measured by Western (1D and G).

Reply: we speculate that analyzying the human cardiomyocytes, even with a reduction of approximately 20% of ST8Sia2 at protein level, there is a large impact on the substrate levels as measured using the polysialic acids (polySia) due to the reduced enzyme. We believe that the ST8Sia2 enzyme has low turnover due to its localization and physicochemical properties. However, we tried to retrieve the supplementary data of manuscripts that measured the protein turnover using mass spectrometry, but we were not able to retrieve this information for the ST8Sia2. Analyzying the SH-SY5Y, the effect is clearer comparing the enzyme and substrate levels.

4. The data are clearer in Fig 2 using SH-SY5Y cells instead of iPSc derived cells. However, SH-SY5Y cells are neuroblastoma cells (selected for their propensity to express polysialic acid, presumably(?)) but does infection of neurones occur in natural T. cruzi infections? What is the physiological basis for choosing this cell line?

Reply: we used SH-SY5Y cells since it has higher levels of ST8Sia2, so it would be a good model to manipulate its expression and the effect on *T. cruzi* infection. Of note, it has been previously demonstrated that patients with Chagas disease (CD) in their chronic phase may present severe neurological manifestations (doi: 10.1179/016164110X12644252260637), a factor that results in elevated morbimortality rates. Furthermore, the presence of *T. cruzi* amastigote forms in the central nervous system (CNS) of mice has already been reported, specifically during the chronic phase of *in vivo* experimental infection (doi: 10.4269/ajtmh.2001.65.152). It is worth emphasizing that the parasite *T. cruzi* capability to infect and proliferate in peripheral glial cells (DOI: 10.1590/0074-0276130492) and brain astrocytes (doi: 10.1016/s0361-9230(00)00326-9) of mice has been demonstrated in previous studies.

5. Also in Fig. 2 B the variance (by eye) of signals to b-actin look wider than those on the plots. For example, the third uninfected sample b-actin signal is much stronger than the other two yet the ST8Sia2, PSA-NCAM and poly-Sia and NCAM1 signals look to be lower than the other two. All signals relative to b-actin in infected cells certainly look lower. However, if a large proportion of cells are infected (? – see point 1) why not perform this (for St8Sia2 and NCAM) by quantitative proteomics, an area of expertise in the author's lab?

Reply: we have rechecked the quantitation levels of the replicates in the WB and confirmed their values and their standard deviation. We have performed a large-scale quantitative proteomics experiment on human fibroblasts (unpublished data) and SH-SY5Y cells (Figure 7, new version of

the manuscript), both infected with *T. cruzi,* and we were not able to detect ST8Sia2 and NCAM using our current mass spectrometry platform, probably due to their low expression/membrane location.

6. *Is the reduction in NCAM due to reduced protein stability or reduction in rate of protein synthesis? A pulse-chase experiment might resolve this issue.*

Reply: we have raised this hypothesis and discussed in the text (lines 789 - 809, new version of this manuscript with track changes on). In brief, it's possible that the reduced levels of polysialylation could affect NCAM protein stability and, consequently, its expression.

7. *The data in Fig 3 with SH-SY5Y cells using anti-ST8Sia2 and anti-PSA immunofluorescence and EndoN-releases PSA measures are striking and seem to be mich more profound that the data in Fig 2 – why is that? Further, the uninfected micrograph seems to have a small number od cells with strong signal but most at background level closer to the signals seen in the infected cells micrograph (again what 5 cells are infected with how many amastigotes?)*

Reply: we believe that using different techniques although all of them are pointing towards a reduction of PSA, there is some variation in the extent of this change. For example, the antibody-based approach might have some binding background which is absent in the HPLC-based approach. Furthermore, up to Figure 3, the summarized results were planned/designed to experimentally demonstrate that *T. cruzi* infection affects host polysialylation. To achieve this, we employed immunofluorescence and HPLC-approach to corroborate the findings highlighted with Western blotting (WB). The purpose of these experiments was solely to demonstrate through different techniques that the downregulation phenotype of ST8Sia2, and consequently, polySia biosynthesis, is modulated in the presence of *T. cruzi* parasites.

8. *In Fig 3, where did the pre-existing PSA of the infected cells go to?*

Reply: we believe that the lower levels of polySia are due to lower levels of ST8Sia2. Indeed, lower levels of enzyme will reduce the addition of polySia to their substrates, due to that we observed both in IF and HPLC, lower levels of polySia.

9. *The removal of cell surface PSA with EndoN makes the cells more easily invaded and/or allows that parasites to propagate better than in control cells. Since the only parameter reported is average number of amastigotes per cell it is impossible to judge which. Again, % infected cells and parasites/cell with SD are needed to assess the data.*

Reply: In the new version of figure 2, we showed the percentage of infected and parasites/cell in SH-SY5Y cells. Additionally, we also demonstrated the same paraments in SH-SY5Y cells treated with CMP (chemical inhibitor for ST8Sia2) and si*ST8Sia2* SH-SY5Y cells infected by *T. cruzi,* as showed in the new version of figure 5.

10. The data in Fig 5 are fascinating in principle but they lack important controls. We are asked to assume that CMP and ST8Sia2 siRNA do indeed reduce cell surface PSA (for some reason, that data is not presented using the techniques in the prior experiments) and that this, and this alone, is the reason for the increase in cell infection [Again, % infected cells and parasites/cell with SD are needed to assess the data, not just average amastigotes per cell]. Note: this reviewer is sceptical that exogenously added CMP would provide selective inhibition of Golgi enzyme ST8Sia2 (or even be taken up by cells at all). The authors should design experiments (perhaps more using EndoN?) where they can correlate extent of PSA reduction with infection efficiency.

Reply: It has been reported that CMP treatment reduces expression of polySia in IMR-32 cells (Figure 3 and Figure 4b *vs.* Figure 4e, as reported in the article 10.1371/journal.pone.0073366). Moreover, it was shown that in CHP-134 cells, a neuroblastoma cell line similar to SH-SY5Y, silencing of ST8Sia2 reduces the levels of polySia. We have included in the manuscript a sentence stating the need of more data on the direct effect of CMP on the inhibition of Golgi ST8Sia2. However, our siRNA experiment shows that lower levels of this enzyme favour the intracellular parasite survival. In addition, we quantified the polySia levels in SH-SY5Y after *ST8Sia2* silencing (results presented in figure 3S). Additionally, we also demonstrated using IF technique that the *ST8Sia2* silencing in the SH-SY5Y cells provides notable reduction in the polySia levels (results can be seen in the figure 3S).

Reviewer #2: The work of Barboza et al. contains important information regarding the modulation of trypanosoma cruzi of host machinery during infection and will be a great contribution in the area if published by PNTD.

The authors performed fine experiments associating infection, enzyme expression and acceptors of the transferred sugars, showing an inverse relationship between enzymatic activity and host polysialylation and infection load. To reach more general and conceptual conclusions, I will address a few points.

1. It was very convincing to use two different lines of cells to see the effect of the infection and the phenotype of the cell, it would also be interesting to see two polar strains of t cruzi, one more and one less invasive to determine if there is a correlation with the presence of the enzymatic activity of ST8Sia2 and the products detected.5CN5A.

Reply: we agree with reviewer 2 on the need to assess this effect in other discrete typing units (DTUs), considering that the DTUs are essential for various aspects of *T. cruzi* infection, including the dynamics of host infection, clinical relevance, and evolutionary history of *T. cruzi* parasites. However, we opted for leaving this experiment for future studies. we have raised this hypothesis and discussed in the text (described between lines 924 - 997, new version of this manuscript with track changes on).

2. Decreased RNAI expression of ST8sia2 produced an increase in infectivity of T cruzi, would the opposite situation of overexpression of the enzyme produce a decrease in infection?

Reply: we agree with the reviewer, but we are not able to perform the overexpression of this enzyme. On the contrary we have deepened our understanding on the potential mechanism on the role of ST8Sia2 during *T. cruzi* infection.

3. The authors do not work on or speculate what is happening in the modulation by the parasite. Parasitic extracts, fixed parasites, alteration of parasite surface, blockade of invasion by antibodies have impact on the enzymatic activity of ST8Sia2.

Reply: We would like to thank #Reviewer2 for the suggestions. We find them quite insightful and believe they could yield promising results. However, based on the results shown in Supplementary Figure 1 (S1 Fig), transcripts modulation of *ST8Sia2* in response to *T. cruzi* infection only occurs at later stages of infection, suggesting that polysialylation may have more direct roles in the parasite's intracellular survival, with little impact during the invasion process. This hypothesis is further supported by the findings presented in Figures 2 and 5, where SH-SY5Y cells with silenced *ST8Sia2* (Figure 5D, E, and G) infected with *T. cruzi* after 48 h.p.i. exhibit a higher number of intracellular parasites compared to non-silenced cells (Figure 2 B). Additionally, we have provided evidence that modulation of ST8Sia2 impacts the cellular oxidative stress favoring a permissive environment for *T. cruzi*.
